# Long Noncoding Competing Endogenous RNA Networks in Pancreatic Cancer

**DOI:** 10.3389/fonc.2021.765216

**Published:** 2021-10-25

**Authors:** Guangbing Xiong, Shutao Pan, Jikuan Jin, Xiaoxiang Wang, Ruizhi He, Feng Peng, Xu Li, Min Wang, Jianwei Zheng, Feng Zhu, Renyi Qin

**Affiliations:** Department of Biliary-Pancreatic Surgery, Tongji Hospital, Tongji Medical College, Huazhong University of Science and Technology, Wuhan, China

**Keywords:** pancreatic cancer, long noncoding RNA, microRNA, competing endogenous RNA, network

## Abstract

Pancreatic cancer (PC) is a highly malignant disease characterized by insidious onset, rapid progress, and poor therapeutic effects. The molecular mechanisms associated with PC initiation and progression are largely insufficient, hampering the exploitation of novel diagnostic biomarkers and development of efficient therapeutic strategies. Emerging evidence recently reveals that noncoding RNAs (ncRNAs), including long ncRNAs (lncRNAs) and microRNAs (miRNAs), extensively participate in PC pathogenesis. Specifically, lncRNAs can function as competing endogenous RNAs (ceRNAs), competitively sequestering miRNAs, therefore modulating the expression levels of their downstream target genes. Such complex lncRNA/miRNA/mRNA networks, namely, ceRNA networks, play crucial roles in the biological processes of PC by regulating cell growth and survival, epithelial–mesenchymal transition and metastasis, cancer stem cell maintenance, metabolism, autophagy, chemoresistance, and angiogenesis. In this review, the emerging knowledge on the lncRNA-associated ceRNA networks involved in PC initiation and progression will be summarized, and the potentials of the competitive crosstalk as diagnostic, prognostic, and therapeutic targets will be comprehensively discussed.

## Introduction

Pancreatic cancer (PC) is a highly aggressive malignancy with a dismal prognosis and limited treatment options worldwide (1). According to cancer statistics, it is the fourth leading cause of cancer-related deaths in the USA, with an overall 5-year survival rate of 8% and a median survival time of 6 months (2). Patients are often asymptomatic, and approximately 80%–85% of PC patients have unresectable or metastatic lesions at the time of initial diagnosis. Surgical resection remains the exclusive potential curative treatment. Owing to the aggressive nature of this neoplasm, early postoperative recurrence and occult metastasis also reduce the efficacy of surgical treatment, and only approximately 20% of patients treated with postoperative adjuvant chemotherapy can survive for 5 years (3). Systemic chemotherapy is indispensable in the treatment of advanced or metastatic PC. Despite many attempts to optimize the chemotherapeutic regimens for PC in clinical studies, such as FOLFIRINOX (fluorouracil, leucovorin, oxaliplatin, and irinotecan), gemcitabine/Nab-paclitaxel, gemcitabine/erlotinib, gemcitabine/capecitabine, and capecitabine/oxaliplatin (XELOX), the increase in the overall survival rate is still poor. By contrast, there is little evidence to support the efficacy of radiotherapy in the treatment of PC (4). Thus, there is an urgent need for a better understanding of the molecular mechanisms of PC to improve patient prognosis.

Although several genes and pathways have been found to be involved in the occurrence and progression of PC, the underlying mechanisms remain unclear. According to previous studies, the mutations of the driver genes in the sentinel cell are the primary cause of tumor initiation (5, 6). These genetic alterations in the oncogene Kirsten RAt Sarcoma virus (*KRAS*) and tumor suppressor genes such as tumor protein 53 (*TP53*), cyclin-dependent kinase inhibitor 2A (*CDKN2A*), and Smad4 together lead to the occurrence of PC (7). *KRAS* mutations, which occur in more than 90% of PCs, are one of the most frequent oncogene changes associated with PC development (8, 9). Subsequently, at the later stage compared with the *KRAS* mutation, the inactivation of *TP53*, *CDKN2A*, and *Smad4* plays a key role in the pathogenesis and invasion of PC (10). Accumulating studies have revealed that the disorders of various signaling pathways mediate changes in the tumor stromal cells, and this process is closely associated with the occurrence and progression of PC (11, 12). Mutations in *KRAS* and epidermal growth factor receptor (EGFR) can activate different signaling pathways including renin-angiotensin system/rapidly accelerated fibrosarcoma/Mitogen-activated protein kinase kinase/extracellular-signalregulated protein kinase (Ras/Raf/MEK/ERK) and phosphatidylinositol-3-kinase (PI3K)/Akt (13, 14). In recent studies, targeting and regulating the key signaling molecules in these pathways have become a hot research topic for improving PC therapy (13). In addition, during the progression of PC, there are dysregulations of important signaling pathways such as EGFR/mitogen-activated protein kinase (MAPK), tumor necrosis factor-related apoptosis-inducing ligand/ tumor necrosis factor receptor associated factor 2 (TRAIL/TRAF2), and Ikappa B kinase/nuclear factor-κ-gene binding (IKK/NF-κB), and in these signaling pathways, not only the apoptosis-inhibiting related proteins but also the expression of many other molecules including B-cell lymphoma-2 (Bcl-2), baculoviral IAP repeat containing 5/(BIRC5), inhibitor of apoptosis protein 3 (IAP3), and cellular inhibitor of apoptosis protein (cIAP) has changed (15). At present, the research on PC-related pathways has become more attractive.

According to previous reports, most RNAs do not encode proteins (16). These noncoding RNAs (ncRNAs) can be divided into long noncoding RNAs (lncRNAs), circular RNAs (circRNAs), microRNA (miRNA), enhancer ncRNAs, etc., and are closely related to a variety of malignant tumors including PC (17, 18). With the support of innovative technologies such as high-throughput RNA sequencing, a large number of ncRNAs have been discovered and clearly classified (19). The ncRNAs play important roles in a variety of biological processes and have regulatory function in the process of transcription and posttranscriptional gene expression (20). The dysregulation of ncRNAs affects many cellular processes including signal transduction, posttranscriptional modifications, and chromatin remodeling, which is closely related to the occurrence and development of various cancers (21, 22). In addition to acting as tumor suppressors or oncogenic driver genes in a variety of malignant tumors, ncRNAs also regulate various molecules in the signaling pathways to exert effects (23, 24). The aberrant expression of ncRNAs participates in the regulation of drug resistance, cell invasion, metastasis, and other processes, which ultimately affects the development of PC (25–27). There are also interactions between different RNAs, and studying the interactions of RNAs may be helpful for the further understanding of PC pathogenesis (28, 29). In recent years, studies have reported the correlation between the aberrant expression levels of different ncRNAs in PC, including the interaction between mRNA and ncRNAs, basing on the competing endogenous RNAs (ceRNA) hypothesis (29, 30).

## The ceRNA Hypothesis in Cancers

MiRNAs are short endogenous RNAs with a length of approximately 21–23 nt (31). The binding sites of miRNAs are called miRNA recognition elements (MREs), which are most commonly found in the 3′-untranslated regions (3′-UTRs) of RNA transcripts such as mRNA (32). In the traditional concept, miRNAs, as the regulatory molecules of the gene expression, bind to the MREs on the mRNAs and then guide the Argonaute protein to the target mRNA, leading to mRNA degradation or gene expression inhibition (33, 34). However, with the further in-depth research on RNA interaction, Franco-Zorrilla et al. (35) have discovered that ncRNAs could relieve the inhibitory effect of miR-399 on its target RNA in plants. Furthermore, Ebert et al. (36) found the similar molecular effects in the animal experiments. Studies indicate that miRNAs are regulated by other ncRNAs bearing MRE sequences in the process of regulating mRNA gene expression (37, 38). Different ncRNAs may possess the same MRE sequence, so multiple ncRNAs may competitively bind to the same miRNA (39). Initially, the phenomenon that ncRNAs compete with mRNAs to bind miRNAs through MREs is called “RNA sponge” (40). In 2011, Salmena et al. (41) formally proposed the ceRNA hypothesis, calling such ncRNAs that competitively bind miRNAs as ceRNAs.

It has been reported that miRNAs could be regulated by various RNA molecules such as lncRNA, circRNAs, and pseudogenes (37). There are over 500 miRNA genes in the human genome, and more than half of mRNA genes may carry MREs (42–44). Multiple miRNAs can regulate a single RNA with various MREs, while multiple RNAs may contain the same specific MRE (34). The different types of these RNA interactions together constitute ncRNA–miRNA–mRNA ceRNA regulatory networks. Current research indicates that the concentration of ceRNAs and miRNAs affects the competition efficiency of the ceRNA–miRNA network (45). In addition, RNA-binding proteins (RBPs), RNA 3′-UTRs, and the subcellular localization of ceRNAs all affect the activity of ceRNAs (36, 45). According to statistics, the potential targets of miRNAs account for more than 60% among the genes that encode human proteins (41, 46). Therefore, changes in the influencing factors of ceRNAs can lead to the imbalance of the ceRNA networks, which may further contribute to the occurrence or development of diseases, including cancer (47).

After the ceRNA hypothesis was put forward, more and more studies, supported by bioinformatics and other technologies, have confirmed the existence of ncRNA–miRNA–mRNA regulatory networks in cancer (29, 47). Researchers have discovered dense MREs in most cancer-related coding genes and lncRNAs in the human genome (32). In cancer cells, these aberrantly expressed lncRNAs interact and further affect the expression of miRNAs through the ceRNA network, which ultimately regulate related cancer genes (32). The lncRNA highly upregulated in liver cancer (HULC) was found to inhibit miR-372 as a ceRNA in liver cancer, thereby increasing the expression of cAMP-dependent protein kinase catalytic subunit beta (PRKACB) (48). In non-small cell lung cancer, LINC81507 acts as the sponge of miR-199b-5p and exerts effects through the Caveolin1/signal transducer and activator of transcription-3 (CAV1/STAT3) signaling pathway (49). In addition, the ceRNA network formed by lncRNA–miRNA–mRNA plays an important role in various cancers including PC. The lncRNA/miRNA/mRNA ceRNA networks in PC are shown in **Supplementary Table 1**.

## LncRNA-Mediated CeRNA in Pancreatic Cancer Pathogenesis and Development

**Figure 1:**
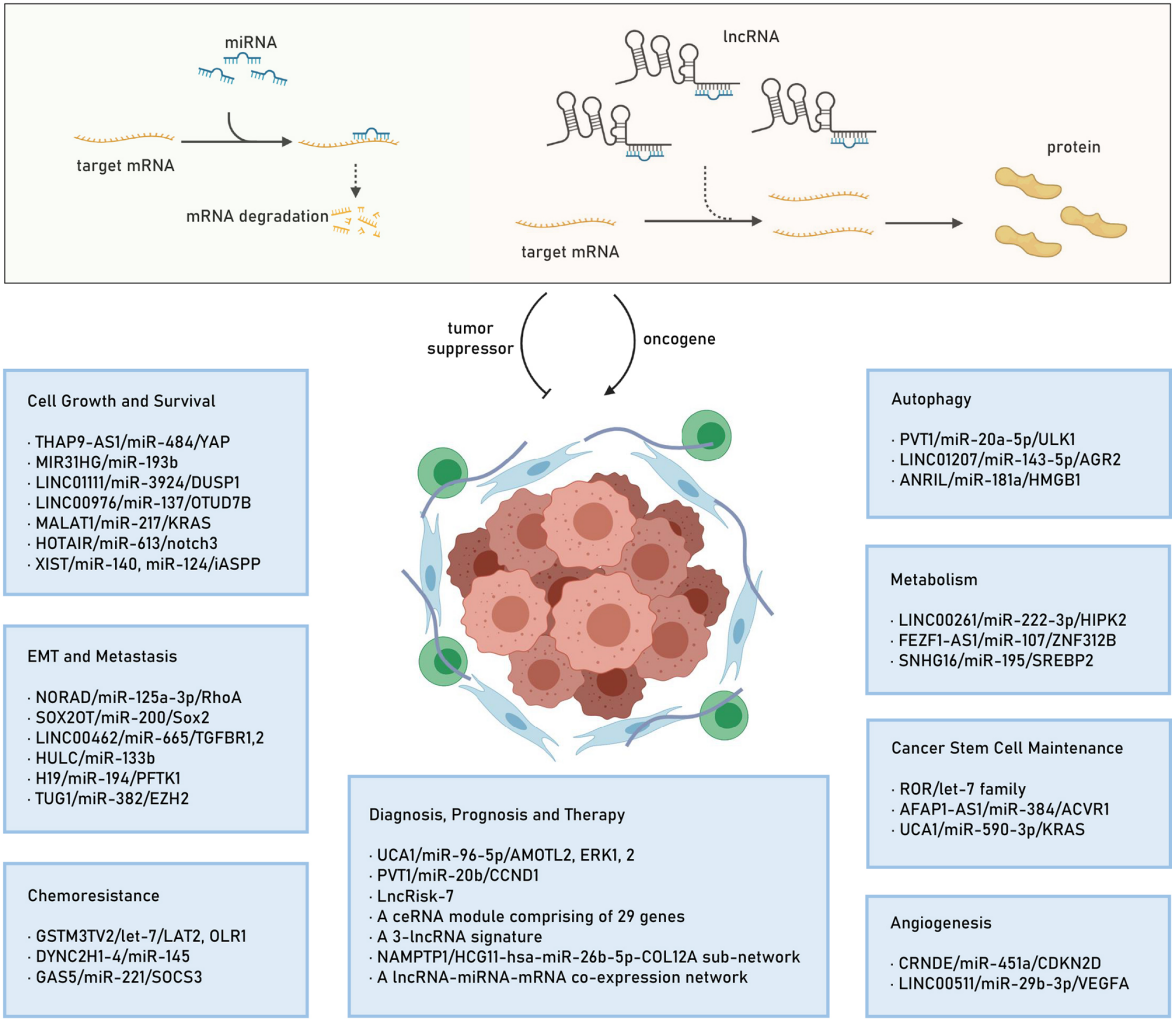
The lncRNA mediated ceRNA mechanism and the identified lncRNA/miRNA/mRNA networks in several hallmarks of PC. lncRNA, long noncoding RNA; ceRNA, competitive endogenous RNA; miRNA, microRNA; mRNA: mRNA, messenger RNA; PC, pancreatic cancer; EMT, epithelial-mesenchymal transition.

Recently, mounting evidence indicates that the identified lncRNAs could exert their oncogenic roles by acting as ceRNAs to regulate target gene expression (20, 50–53), thereby modulating cell proliferation (54), apoptosis (55), cell cycle (56), invasion and metastasis (57), epithelial–mesenchymal transition (EMT) (58), metabolism (59), autophagy (60), angiogenesis (61), stemness (62), as well as chemoresistance (63), thus involving in PC pathogenesis and progression (64–67). In this section, we will elucidate the functions of some lncRNA-mediated ceRNA regulatory networks in PC. Also, we highlight the ceRNA regulatory networks consisting of an lncRNA/miRNA/mRNA axis. We summarize the identified lncRNA/miRNA/mRNA networks in several hallmarks of PC in **Figure 1**.

## LncRNAs as ceRNAs Regulating Cell Growth and Survival

Cell growth and survival are complicated processes (68, 69) that are tightly regulated by tumor suppressor genes, oncogenes, along with other controlling mechanisms and associated with the hallmarks of sustaining proliferative signaling, evading growth suppression, enabling replicative immortality, and resisting cell death (65, 68, 70). Recent studies have uncovered the regulatory role of lncRNAs in cell growth and survival through multiple mechanisms in PC (20, 53, 65–67, 71). Apart from the oncogenic role of lncRNAs, growth and survival are also regulated by several lncRNA-mediated ceRNA networks in PC (67, 71). In this section, we will discuss some ceRNA networks and their role in PC cell growth and survival.

## THAP9-AS1/miR-484/YAP

LncRNA THAP domain containing 9 antisense RNA 1 (THAP9-AS1), which is an antisense transcript of THAP9 and locates at chromosome 4q21.22, has been reported to act a key role in the tumorigenesis of gastric cancer (72) and esophageal squamous cell carcinoma (73, 74). A recent study by Li et al. (54) demonstrated that THAP9-AS1 promoted the cell growth of PC through the THAP9-AS1/miR-484/yes-associated protein (YAP) ceRNA pathway. Clinical evidence showed that THAP9-AS1 was overexpressed in PC tissues and significantly associated with poor prognosis of patients. THAP9-AS1 promoted PC cell growth both *in vitro* and *in vivo*. Ectopic THAP9-AS1 expression bound to miR-484 directly, and such competitive binding decreased the abundance of miR-484 and relieved its repression of the downstream target, YAP, an important downstream nuclear effector of the Hippo signaling pathway. Inversely, YAP overexpression or knockdown diminished the effects of THAP9-AS1 modulated in PC cells. Moreover, THAP9-AS1 bound to YAP protein and inhibited the phosphorylation-mediated inactivation of YAP by large tumor suppressor kinase 1 (LATS1). Reciprocally, YAP bound to THAP9-AS1 promoter *via* transcriptional enhanced associate domain 1 (TEAD1) and promoted THAP9-AS1 transcription to form a positive feedback regulatory loop in PC cells. Importantly, THAP9-AS1 level positively correlated with YAP expression in PC tissues. Thus, THAP9-AS1/miR-484/YAP axis might serve as a potential biomarker and therapeutic target for PC treatment.

## MIR31HG/miR-193b

LncRNA miR-31 host gene (MIR31HG) is a recently identified 2,166-nt lncRNA and regulated by methylation of the promoter region in transcription level (75, 76). Accumulating studies have revealed that MIR31HG plays oncogenic or tumor-suppressive roles in cancer initiation and progression (75), and its overexpression can serve as a prognosis predictor for several malignancies, including oral cancer (77), hepatocellular carcinoma (78), and head and neck squamous cell carcinoma (79). Yang et al. (55) demonstrated that MIR31HG was markedly upregulated in PC tissues and cell lines. Knockdown of MIR31HG significantly suppressed PC cell growth, promoted apoptosis, and induced cell cycle G1/S arrest, whereas enhanced expression of MIR31HG exerted the opposite effects. Mechanistically, MIR31HG acted as an endogenous sponge by competing for miR-193b and regulated miR-193b targets, such as cyclin D1 (CCND1), myeloid cell leukemia sequence 1 (Mcl-1), ecto-5'-nucleotidase (NT5E), KRAS, u-plasminogen activator (uPA), and E-twenty six transcription factor 1(ETS1). Meanwhile, inhibition of miR-193b expression significantly upregulated the MIR31HG level, while overexpression of miR-193b suppressed MIR31HG’s expression and function in PC cells. As a result, these results demonstrated that MIR31HG functioned as an oncogene to promote tumor progression, and MIR31HG/miR-193b axis served as a potential therapeutic target for PC (55).

## LINC01111/miR-3924/DUSP1

LncRNA LINC01111 is a novel long intergenic ncRNA and located at chromosome 8q21.13 (80, 81). Pan et al. (81) found that LINC01111 expression was significantly downregulated in PC tissues and plasma and was positively associated with lymph node metastasis and tumor stage. Lower expression of LINC01111 was correlated with poor prognosis in PC patients. LINC01111 overexpression significantly inhibited cell proliferation and induced cell cycle G1/S arrest *in vitro*, as well as tumorigenesis *in vivo*. Conversely, LINC01111 knockdown enhanced cell proliferation and promoted cell cycle G1/S transition *in vitro*, as well as tumorigenesis *in vivo*. Meanwhile, the results also demonstrated that LINC01111 functioned as a molecular sponge for miR-3924 to upregulate dual-specificity protein phosphatase 1 (DUSP1) protein levels and then downregulate stress-activated protein kinase (SAPK) phosphorylation and the translocation of p-SAPK from the cytoplasm to the nucleus. Thus, the loss of LINC01111 in PC activated the SAPK/c-Jun N-terminal kinase (JNK) signaling pathway, resulting in the promotion of tumor growth. Moreover, LINC01111 also facilitated an important role in PC cell invasion and metastasis. Collectively, this study indicated that LINC01111/miR-3924/DUSP1 axis was a potential therapeutic target for treating PC (81).

## LINC00976/miR-137/OTUD7B

LncRNA LINC00976, a novel long intergenic ncRNA, has been recently identified as an oncogenic lncRNA to promote the cell growth of PC through the LINC00976/miR-137/ovarian-tumour family deubiquitinases domain-containing protein 7B (OTUD7B) (Cezanne) ceRNA pathway (82). The data showed that LINC00976 expression was overexpressed in PC tissues and cell lines and was positively associated with poorer survival in patients with PC. Function studies revealed that LINC00976 knockdown significantly suppressed cell proliferation, migration, and invasion *in vivo* and *in vitro*, whereas its overexpression reversed these effects. Furthermore, bioinformatics analysis, luciferase assays, and rescue experiments revealed that LINC00976/miR137/OTUD7B established a ceRNA network to modulate PC cell proliferation and tumor growth. Ultimately, OTUD7B mediated EGFR and MAPK signaling pathway, which suggested that LINC00976/miR-137/OTUD7B/EGFR axis might act as a potential biomarker and therapeutic target for PC (82).

## MALAT1/miR-217/KRAS

LncRNA metastasis-associated lung adenocarcinoma transcript 1 (MALAT1), which is an evolutionarily highly conserved lncRNA and localized on chromosome 11q13, has been shown to be involved in the pathogenesis of multiple cancers by acting as an oncogene to promote cell growth, evade apoptosis, regulate cell cycle, maintain stemness, and enhance invasion and metastasis (83–85). Moreover, it is significantly overexpressed in many cancer types and may be related to tumor prognosis, indicating its potential use as a biomarker of cancers (83, 85). MALAT-1 played an important role in the carcinogenesis of PC by acting as a ceRNA. Liu et al. (86) demonstrated that MALAT1 functioned as a molecular sponge for miR-217 to upregulate the expression of KRAS for promoting tumor growth in PC. Knocking down MALAT1 reduced pancreatic tumor cell growth and proliferation both *in vitro* and *in vivo*. And MALAT1 knockdown also inhibited cell cycle progression and impaired tumor cell migration and invasion. However, MALAT1 knockdown attenuated the protein expression of KRAS not directly through inhibition of cellular miR-217 expression but decreased the miR-217 nucleus/cytoplasm ratio, which suggested that MALAT1 inhibited the translocation of miR-217 from the nucleus to the cytoplasm. Thus, MALAT1 acted as a tumor promoter at least in part by binding miR-217 and sequestering the molecule in the nucleus, thereby promoting oncogenic KRAS expression in PC (86). In contrast to the previous study by Liu et al., another study confirmed that the MALAT1 suppressed miR-200c-3p function *via* upregulating zinc finger E-box-binding homeobox 1 (ZEB1) expression to induce the capability of PC cell migration and invasion (87). Therefore, it can be proposed that MALAT1 could be a potential therapeutic target in PC.

## HOTAIR/miR-613/notch3

LncRNA HOX transcript antisense RNA (HOTAIR), which is a well-characterized oncogenic lncRNA and dysregulated in variety of cancers, localizes in the HOXC locus of chromosome 12q13.13 that flanks between HOXC11 and HOXC12 loci (88–90). Notably, a growing body of evidence suggests that HOTAIR constitutes a critical contributor to various known or unknown mechanisms in the pathogenesis and progression of multiple cancers and is also an important negative prognostic factor for cancer patients, including PC (71, 88–90). Cai et al. (91) demonstrated that HOTAIR could act as a ceRNA *via* regulating miR-613/notch3 axis to promote cell growth and survival in PC. They revealed that HOTAIR was found to be upregulated in both PC tissues and cell lines, and HOTAIR was inversely correlated with miR-613 level in PC tissues. Knockdown of HOTAIR in PC cells suppressed the expression levels of miR-613 and tumor growth, suggesting that the oncogenic role of HOTAIR might be correlated with miR-613. Further investigation confirmed that HOTAIR suppressed miR-613 expression *via* sponging miR-613 in the PC cells. Thus, the HOTAIR/miR-613/notch3 axis might be a promising therapeutic target for PC (91). Meanwhile, Deng et al. (92) reported that HOTAIR sponged miR-34a to promote PC stem cell-like properties through activation of the JAK2/STAT3 pathway. Silencing of HOTAIR could inhibit the Wnt/β-catenin signaling pathway to alleviate EMT in PC (93).

## XIST/miR-140, miR-124/iASPP

LncRNA X inactivation-specific transcript (XIST) is derived from XIST gene and is important for inactivation of X chromosome in the development of female mammals (94). It is reported that XIST is dysregulated in a variety of cancers and exerts its either tumor-suppressive or oncogenic role in tumorigenesis and progression of cancers, such as hepatocellular carcinoma, lung cancer, gastric cancer, and osteosarcoma (95–97). Recent studies indicated that XIST was overexpressed in PC and involved in regulating the cell proliferation, apoptosis, migration, and invasion (98). Liang et al. (56) demonstrated that XIST was specifically upregulated in PC tissues and related to the advanced TNM stage and larger tumor dimension. Patients with high XIST expression correlated to poorer survival compared with that low expression. Knockdown of XIST could induce PC cell cycle arrest at G0/G1 phase by regulating cell cycle arrest-related CDK1 and P21, and p53-independent apoptosis-related factor iASPP, which significantly leads to suppression of the cell viability and proliferation *in vivo* and *in vitro*. Mechanistically, XIST functioned as a ceRNA for interacting with miR-140 and miR-124 to upregulate the inhibitor for the apoptosis-stimulating protein of p53 (iASPP) expression. Meanwhile, iASPP could suppress p73 transcriptional activity to decrease the inhibitory effect of p73 on XIST expression without changing p73 protein levels. Moreover, XIST was inversely correlated with miR-140, miR-124, and p21, respectively, and positively correlated with iASPP and CDK1. Thus, these data all indicated that XIST played a key role in regulating PC cell proliferation and cell cycle and might provide a potential therapeutic strategy for PC (56). In addition, it has been proven that XIST/miR-133a/EGFR (99), XIST/miR-34a-5p (98), and XIST/miR-137/Notch1 (100) ceRNA axes also played important roles in PC cell growth and survival regulation, while XIST/miR-429/ZEB1 (101) and XIST/miR-141-3p/TGF-β2 (102) ceRNA axes contributed to PC cell migration and invasion.

## LncRNAs as ceRNAs Affecting Epithelial–Mesenchymal Transition and Metastasis

EMT is a complex and developmental process in which polarized epithelial cells lose their characteristics instead of acquire mesenchymal properties with the capacity of migration and metastasis, playing a critical role in the progression of cancers (65, 68, 103–105). It has been shown that epithelial cells in this process that are induced by the transcriptional factors Snail, Twist, Slug, ZEB1, and ZEB2, would result in loss of E-cadherin expression and acquisition of mesenchymal markers, such as N-cadherin or vimentin (106–108). Recent studies have indicated that lncRNAs regulate PC EMT and metastasis (20, 53, 109, 110), and therefore, the mechanism underlying the role of lncRNAs should be addressed, knowing that some lncRNAs may serve as ceRNA for PC EMT and metastasis.

## NORAD/miR-125a-3p/RhoA

LncRNA noncoding RNA activated by DNA damage (NORAD, also known as LINC00657) is a highly conserved, ubiquitously expressed cytoplasmic lncRNA and locates on chromosome 20q11.23, which is required for maintaining chromosomal stability and proper mitotic divisions in human cells (111, 112). Recent evidence indicates that NORAD is dysregulated in various human cancers and acts as an important regulator by interacting with different types of mechanisms to promote tumor progression, such as cell proliferation, invasion, metastasis, and apoptosis (113, 114). Chen et al. (58) revealed that NORAD could enhance the hypoxia-induced EMT to promote PC cell metastasis by acting as a ceRNA. Notably, they firstly revealed that NORAD expression was highly increased in PC tissues by using human microarray datasets GSE15471 and GSE16515 for analyzing its expression profile, and NORAD expression was significantly upregulated after hypoxic stimulation for 48 h. Knockdown of NORAD impaired PC cell migration and invasion *in vitro* and decreased the metastatic and disseminated ability in an orthotopic mouse metastatic model. Western blotting also showed that knockdown of NORAD significantly suppressed the expression levels of the mesenchymal cell markers N-cadherin, vimentin, and ZEB1 but increased the expression levels of the epithelial cell marker E-cadherin. Furthermore, they demonstrated that NORAD utilized its oncogenic role by directly binding to miR-125a-3p and inhibiting its expression in PC cells, thus leading to upregulation of RhoA expression. Meanwhile, treating with ras homolog gene family (RhoA) pathway specific inhibitor CCG-1423 could impede the flow of EMT and invasive behaviors induced by NORAD. Additionally, patients with higher NORAD expression had shorter overall survival and recurrence-free survival rates. Thus, NORAD/miR-125a-3p/RhoA axis might be a potential novel therapeutic target for the treatment of PC (115). Moreover, Bi et al. (116) also found that lncRNA LINC00657 (NORAD) enhanced PC cell proliferation, migration, and invasion but restricted the apoptosis by acting as a ceRNA on miR-433 to increase protein activated kinase 4 (PAK4) expression.

## SOX2OT/miR-200/Sox2

LncRNA SOX2 overlapping transcript (SOX2OT), which is a highly expressed lncRNA in embryonic stem cells and maps to human the chromosome 3q26.3 locus, plays critical roles in embryogenesis, cell differentiation, and pluripotency maintenance (117). SOX2OT harbors SOX2 gene transcription in its intronic region and produces at least eight transcript variants to exploit its effect on various diseases, including cancer (117, 118). Recent studies have demonstrated that SOX2OT is overexpressed in many cancers and involved in tumor development and progression by acting as an oncogene to promote cell proliferation, invasion, migration, and growth and suppress cell apoptosis (118). Zhang et al. (119) demonstrated that SOX2OT was overexpressed in PC tissues and significantly correlated with TNM staging, acting as a potential prognosis marker for patient outcome. They found that the tumor suppressor YY1 bound to the promoter of SOX2OT and inhibited tumor growth *in vivo* and *in vitro* by suppressing SOX2OT and SOX2 expression in PC. Furthermore, they observed that SOX2OT could promote PC cell EMT by acting as a ceRNA (120). They found that plasma exosomal SOX2OT expression was high and correlated with TNM stage and overall survival rate of PC patients. Further research showed that SOX2OT or exosomal SOX2OT promoted PC cells metastasis and regulated EMT properties by increasing the expression levels of the mesenchymal cell markers N-cadherin and vimentin but suppressing the expression levels of the epithelial cell marker E-cadherin. Mechanistically, SOX2OT competitively bound to the miR-200 family to increase the expression of Sox2, thus promoting invasion and metastasis of PC *in vitro* and *in vivo*. Besides, they also found that SOX2OT/miR-200/Sox2 ceRNA axis could enhance stem cell-like properties of PC (120). Thus, SOX2OT/miR-200/Sox2 played important roles in tumor progression and might be a useful marker for PC prognosis.

## LINC00462/miR-665/TGFBR1, TGFBR2

LncRNA LINC00462, which contains two exons with approximately 962 nucleotides in length and is located on chromosome 13 according to NONCODE 4.0, is found to promote tumor proliferation, migration, and invasion by regulating the AKT signaling pathway in multiple cancers, including hepatocellular carcinoma and renal cell carcinoma (121, 122). Zhou et al. (123) demonstrated that LINC00462 promoted PC invasiveness through the miR-665/TGFBR1-TGFBR2/SMAD2/3 pathway. They found that the expression level of LINC00462 was significantly higher in tumor tissues and was correlated with large tumor size, poor tumor differentiation, TNM stage, and distant metastasis in PC patients. *In vitro*, LINC00462 promoted PC cell migration and invasion but inhibited cell adhesion. *In vivo*, LINC00462 enhanced PC cell metastasis to lung, liver, and spleen in a mouse xenograft model. LINC00462 also regulated PC cell EMT properties by increasing the expression of intracellular adhesion molecule (ICAM)-1, vimentin, Twist1, matrix metalloproteinase (MMP)2, and MMP9 but decreasing the expression of E-cadherin. Further study showed that LINC00462 acted as a ceRNA to promote the malignant phenotype of PC by sponging miR-665, thus upregulating the expression levels of transforming growth factor beta 1 (TGFBR1) and TGFBR2. Ectopic expression of miR-665 could reverse LINC00462 overexpression-mediated cell migration, invasion, and EMT in PC. In contrast, knockdown expression of miR-665 observed the opposite effects. While LINC00462-mediated cell malignant behavior promotion in PC was also rescued by loss of expression of TGFBR1 and TGFBR2. Furthermore, LINC00462 activated the SMAD2/SMAD3 signaling pathway by increasing the expression levels of p-SMAD2/3 and the nuclear distribution of SMAD2/3, which led to upregulating collagen 1, collagen 3, and fibronectin. Meanwhile, LINC00462 played important roles on cell proliferation and tumorigenesis in PC. Taken together, LINC00462/miR-665/TGFBR1/2 regulatory network might be a potential novel therapeutic target for the treatment of PC (123).

## HULC/miR-133b

LncRNA is highly upregulated in liver cancer (HULC), which is originally identified as the most overexpressed lncRNA in hepatocellular carcinoma, and is located on chromosome 6p24.3 with approximately 500 nucleotides in length and contains two exons (124, 125). Increasing evidence demonstrates that HULC is also dysregulated in other types of cancer and plays essential roles in tumor initiation and progress by promoting different tumorigenic phenotypes, such as cell survival, proliferation, and invasion *in vitro*, as well as tumor growth and angiogenesis *in vivo* (124, 125). Peng et al. (126) found that HULC was overexpressed in PC tissues and associated with tumor size, lymph node metastasis, and vascular invasion. And multivariate analysis showed that HULC expression was an independent prognostic indicator for overall survival and time to recurrence of patients with PC. Knockdown of HULC significantly decreased PC cell ability of proliferation and induced cell cycle arrest at G1/S phase *in vitro*. Zheng et al. (127) further revealed that HULC promoted the proliferation and invasion of PC cells but inhibited apoptosis by being involved in the Wnt/β-catenin signaling pathway. Similarly, HULC downregulated the expression of miR-15a, then activated the PI3K/AKT pathway to enhance PC cell ability of migration and invasion (128). Meanwhile, exosomal HULC could function as ceRNA for contributing to PC cell invasion and migration by regulating EMT (129). Exosomal HULC expression was significantly increased in PC patients’ serum compared to healthy individuals or intraductal papillary mucinous neoplasm patients. Further study showed that knockdown of HULC suppressed PC cell invasion and migration and inhibited the EMT by downregulating N-cadherin, vimentin, and Snail but upregulating E-cadherin *in vitro* and *in vivo*. Meanwhile, exosomal HULC derived from PC cells also promoted cancer cell invasion and migration by inducing EMT. Mechanistically, HULC interacted with miR-133b to alter PC cell invasion and migration, as well as EMT (129). Moreover, HULC and miR-622 *via* transfer by extracellular vesicle regulated PC cell invasion and migration (130). Thus, extracellular vesicle-encapsulated HULC could be a potential circulating biomarker and therapeutic target for PC.

## H19/miR-194/PFTK1

LncRNA H19, which is firstly described as a fetal transcript in mice in 1984, is located on chromosome 11p15.5 and expressed exclusively from the maternal allele (131, 132). Recent studies indicate that H19 is dysregulated in various cancer types and serves as oncogene or tumor suppressor to affect the development and progression of cancer through various mechanisms. For example, H19 enhances invasion and metastasis in bladder cancer, glioma, breast cancer, non-small cell lung cancer, and gastric cancer but suppresses the aggressiveness of hepatocellular carcinoma and prostate cancer (131, 133). Further study demonstrated that H19 acted as a ceRNA to enhance invasion and metastasis by regulating Wnt/β-catenin signaling pathway in PC (134). Sun et al. (134) found that H19 was overexpressed and correlated with distant metastasis, advanced TNM stages, and poor survival in patients with PC. Multivariate analysis revealed that high H19 expression was an independent indicator of poor prognosis. H19 knockdown suppressed PC cell migration and invasion *in vitro*. Subsequently, they demonstrated that H19 promoted PC cell invasion and migration at least partially by increasing [cyclin-dependent kinase 14 (CDK14)] expression through antagonizing miR-194. H19 knockdown significantly reduced the expression of PFTK1, while miR-194 inhibition significantly increased the expression of PFTK1; the suppressive effect of H19 knockdown was partially attenuated by miR-194 inhibition and PFTK1 overexpression. Moreover, H19/miR-194 modulated Wnt/β-catenin signaling by upregulating p-LRP6, Snail but downregulating p-β-catenin to promote PC cell invasion and migration. The expression level of H19 and PFTK1 was positively correlated with each other, while miR-194 was negatively correlated with H19 and PFTK1 in tissue samples. Collectively, the H19/miR-194/PFTK1 ceRNA axis might be a potential novel therapeutic target for PC (134). In addition, the H19/let-7/HMGA2/EMT signaling axis also played important roles on PC metastasis and EMT (135). And H19 could maintain PC cell EMT process and stemness by deriving miR-675-3p that directly targeted SOCS5 then activating the STAT3 pathway (136).

## TUG1/miR-382/EZH2

LncRNA taurine upregulated gene 1 (TUG1), which is originally identified in the genomic screen of taurine-treated mouse retinal cells, is a nucleotide lncRNA sequence localized to chromosome 22q12.2 (137, 138). Recent studies have been indicated that TUG1 is dysregulated in numerous human cancers and acts as an unfavorable predictor of survival for patients with cancer, such as renal cell carcinoma, ovarian cancer, bladder urothelial carcinoma, oral squamous cell carcinoma, esophageal squamous cell carcinoma, hepatocellular carcinoma, and intrahepatic cholangiocarcinoma (137, 138). Zhao et al. (139) revealed that TUG1 was essential for the migration and EMT in PC by serving as a ceRNA. They firstly demonstrated that TUG1 was overexpressed in tumor tissues and correlated with large tumor size, poor tumor differentiation, TNM stage, vascular infiltration, distant metastasis, and overall survival of patients with PC, which indicated that upregulated TUG1 might contribute to the development of PC. Then, knockdown of TUG1 decreased the PC cell migration capacity and the formation of EMT by upregulating E-cadherin, β-catenin but downregulating N-cadherin, vimentin *in vitro*. In contrast, overexpression of TUG1 showed opposite effects. Further study confirmed that TUG1 exerted inhibitory effects on miR-382 expression through functioning as a ceRNA and therefore directly sponging miR-382 in PC. Overexpression of miR-382 could reverse the TUG1 effects on the promotion of PC cell migration and EMT formation. Additionally, TUG1 could positively regulate the expression of EZH2, a target of miR-382, by decreasing miR-382. Knockdown of EZH2 abolished PC cell migration and EMT formation, which was caused by TUG1 overexpression. Moreover, the expression level of TUG1 was negatively correlated with miR-382 and positively correlated with EZH2 in PC tissues. Collectively, these data indicated that TUG1/miR-382/EZH2 ceRNA regulatory signaling pathway enhanced PC cell migration capacity and EMT formation and might be a potential novel therapeutic target for PC (139). Otherwise, TUG1/miR-29c axis was also critical for promoting the growth and migratory ability of PC cells *in vitro* and *in vivo* (140). Inhibition of TUG1/miR-299-3p ceRNA axis suppressed PC cell malignant progression *via* deactivation of the Notch1 pathway (141).

## LncRNAs as CeRNAs Related to Cancer Stem Cell Maintenance

Cancer stem cells (CSCs) are a functional subpopulation of cells that exhibit high proliferation, self-renewal, and high tumorigenic, invasive, and metastatic capability, as well as chemoresistance, and their abundance is positively associated with the degree of PC malignancy (69, 142, 143). Studies have revealed that the cell surface proteins CD44, CD24, CD133, chemokine C-X-C-motif receptor 4 (CXCR4), aldehyde dehydrogenase 1 family, member A1 (ALDH1), Epithelial cell adhesion molecule (EPCAM), adenosine triphosphate binding box transporter G2 (ABCG2), and cellular-mesenchymal epithelial transition factor (c-MET) are identified as PC stem cell markers (69, 143, 144). Several lines of evidence have shown that oncogenic lncRNAs help sustain cancer stem cell traits by acting as ceRNAs in the initiation and progression of PC (20, 53, 145). Thus, the lncRNA-mediated ceRNA network may serve as a potential biomarker and therapeutic target for PC.

## ROR/let-7 Family

LncRNA regulator of reprogramming (ROR), which is highly expressed in induced pluripotent stem cells (iPSCs) and embryonic stem cells (ESCs), is located at 18q21.31 and can be regulated by pluripotency transcription factors, such as Sox2, Oct4, and Nanog (146, 147). It has been identified that ROR is an important regulator of reprogramming differentiated cells to iPSCs and maintenance of ESCs, which indicates that ROR plays critical roles in tumorigenesis and progression of human cancer (146, 147). Accumulating evidence has demonstrated that ROR is upregulated in multiple types of cancer and associated with tumor metastasis, EMT program, drug resistance, and stem cell-like characteristic promotion by various regulatory mechanisms in ovarian, lung, breast cancer, hepatocellular cancer, gastric cancer, and so on (146, 147). Meanwhile, recent studies also reveal that ROR acts as a ceRNA by sponging miR-145 (148), miR-205 (149), and miR-34a (151) to regulate gene transcription. Zhan et al. (150) demonstrated that ROR was overexpressed in PC tissues and enhanced PC cell metastasis, EMT promotion, and tumor growth by activation of ZEB1 pathway. Similarity, another study showed that ROR could modulate the expression of polypyrimidine tract-binding protein 1/pyruvate kinase isozymes M2 (PTBP1/PKM2) through sponging miR-124 to induce PC cell autophagy, which led to gemcitabine resistance for PC (152). Moreover, Fu et al. (153) revealed that ROR functioned as a ceRNA to promote stem cell-like phenotype in PC. They firstly found ROR was significantly upregulated and positively correlated with poor prognosis in patients with PC. Knockdown of the expression of ROR impaired cell proliferation, migration, and invasion ability, suppressed the EMT process, and induced cell cycle G1/S arrest in PC. Further study displayed that ROR was overexpressed in PC stem-like cells and promoted PC stem-like cell sphere formation capability *in vitro* and tumorigenicity *in vivo* by regulating the expression of Sox2 and Nanog. Mechanistically, ROR exerted its oncogenic effects by sponging several tumor suppressor miRNAs such as let-7 family (let-7i-5p, let-7b-5p, let-7e-5p, let-7e-3p, let-7b-3p, and let-7c-3p), miR-93-5p, miR-145-3p, miR-320a, and miR-320b to maintain the cancer stem cell properties of PC. Collectively, ROR was a potential therapeutic target for PC. In addition, Gao et al. (151) showed that ROR/miR-145/Nanog ceRNA axis also contributed to modulate PC cell stem-like properties.

## AFAP1-AS1/miR-384/ACVR1

LncRNA actin filament-associated protein 1 antisense RNA 1 (AFAP1-AS1), which is transcribed from the AFAP1 gene in the antisense direction, is mapped to the 4p16.1 region of human chromosome with 6,810 bp in length (154, 155). AFAP1-AS1 contains several overlapping and complementary regions among the exons of AFAP1-AS1 and can affect the expression of AFAP1. Accumulated studies have shown that AFAP1-AS1 is aberrantly expressed and exerts a carcinogenic role in numerous types of tumors, including breast cancer, liver cancer, gastric cancer, non-small cell lung cancer, and colorectal cancer (154, 155). In PC, AFAP1-AS1 had also been reported to be aberrantly expressed and was able to function as a regulator of tumorigenesis by regulating cell proliferation, apoptosis, migration, invasion, stemness, and so on (156, 157). Wu et al. (62) revealed that AFAP1-AS1 functioned as a ceRNA to regulate the cancer stem cell properties of PC. They first found that AFAP1-AS1 was overexpressed in PC tissues and side population (SP) cells. While SP cells were rich with cancer stem cell markers Oct4, ABCG2, Nestin, CK19, and CD133, which indicated that AFAP1-AS1 was involved in maintaining stemness. Knockdown of AFAP1-AS1 exerted suppressive effects on PC cell sphere formation and clone formation, while overexpression of AFAP1-AS1 group showed the opposite trend. Moreover, AFAP1-AS1 positively regulated the expression of CSC markers Oct4, ABCG2, Nestin, CK19, and CD133 by gain or loss strategies in PC cells. Furthermore, their research identified that AFAP1-AS1 modulated PC cell stemness by upregulating activin receptor type-1 (ACVR1) through competitively binding to miR-384 (62). MiR-384 decreased PC cell ability of sphere formation and clone formation and inhibited the expression of Oct4, ABCG2, Nestin, CK19, and CD133. In contrast, ACVR1 enhanced PC cell stemness by increasing cell sphere formation and clone formation and upregulating of Oct4, ABCG2, Nestin, CK19, and CD133. Their study data also found that AFAP1-AS1-promoted PC cell tumorigenesis and stemness could be reversed by miR-384 *in vivo*. Therefore, these results suggested that AFAP1-AS1/miR-384/ACVR1 pathway might do duty for a potential therapeutic target for PC patients (62).

## UCA1/miR-590-3p/KRAS

LncRNA urothelial cancer-associated 1 (UCA1), a member of the human endogenous retrovirus H family and firstly identified in bladder transitional cell carcinoma, is 1,442 bp in length and located on chromosome 19p13.12 with three exons and two introns (158–160). According to the tissue expression profiling, UCA1 is ubiquitously expressed at post-fertilization primary phase and not expressed in most normal tissues of adults. Further studies have shown that UCA1 is highly expressed and exerts oncogenic activity in numerous cancers, such as gastric cancer, colorectal cancer, liver cancer, breast cancer, cervical cancer, and prostate cancer (158–160). Several studies also indicate that highly expressed UCA1 is related to poor clinicopathological features and may serve as a prognostic marker for cancer patients (161). Meanwhile, an increasing number of studies showed that UCA1 played important roles in tumorigenesis of PC. Chen et al. (162) firstly demonstrated that UCA1 was significantly upregulated in PC and correlated with tumor size, depth of invasion, CA19-9 level, and tumor stage. UCA1 suppressed the expression of P27 to effectively inhibit PC cell proliferative activities, induce apoptotic rate, and cause cell cycle arrest. Zhang et al. (163) revealed that UCA1 promoted cell migration and invasion of PC cells through the Hippo signaling pathway *via* interacting with YAP. Moreover, recent studies have shown that UCA1 promoted progress and development of PC by serving as a ceRNA. Zhang et al. (164) elucidated that UCA1 enhanced PC cell growth, migration, and invasion ability by sponging miR-135a. And a study by Zhou et al. (165) reported that UCA1 could bind miR-96 to modulate the expression of forkhead box protein O3 (FOXO3) that promoted proliferation and metastasis while reduced apoptosis of PC cells. Additionally, Gong et al. (166) discovered that the regulatory network of UCA1/miR-107/ITGA2 regulated the migration and invasion of PC cells through focal adhesion pathway. Besides, Liu et al. (167) found that UCA1/miR-590-3p/KRAS axis was critical for stemness maintenance of PC. They revealed that UCA1 was overexpressed in PC and might be a negative prognostic factor for patients’ overall survival. Knockdown of UCA1 decreased sphere formation capability of PC cells, as well as the expression of the stemness markers CD133, OCT4, NANOG, and SOX2. In contrast, overexpression of UCA1 resulted in the opposite effects. Mechanistically, UCA1 exerted its oncogenic role by enhancing the expression and activity of KRAS. UCA1 firstly could function as a molecular sponge by directly binding to miR-590-3p, which led to upregulating KRAS expression. Then, UCA1 promoted phospho-KRAS protein expression through interaction with hnRNPA2B1 to modulate oncogenic KRAS activity, which was subsequently necessary for tumorigenic activity in PC. Notably, KRAS also significantly promoted UCA1 expression, thus forming a positive feedback loop. Thus, these findings suggested that UCA1/miR-590-3p/KRAS regulatory network might be a target for new PC therapies (167). Meanwhile, Guo et al. (168) demonstrated that UCA1, which is derived from hypoxic PC exosomes, could promote angiogenesis and tumor growth through the miR-96-5p/AMOTL2/ERK1/2 ceRNA axis *in vitro* and *in vivo*.

## LncRNAs as CeRNAs Controlling Metabolism

Metabolism reprogramming has been regarded as a hallmark of cancer (169, 170). As a primary feature in carcinogenesis, metabolic reprogramming contributes to tumor cell proliferation, EMT, metastasis, immune escape, and resistance to chemotherapy (171–173). Meanwhile, reprogramming of cancer metabolism is composed of dysregulation of glucose and glutamine metabolism, alterations of lipid synthesis, rewiring of mitochondrial function, etc. (171–173). Numerous genes have been shown to participate in the regulation of metabolic pathways, thus aberrant expression of these genes can be involved in the pathogenesis of PC (103, 170, 172, 174). The recent studies have revealed a significant attention toward the role of lncRNAs in the regulation of different aspects of cancer metabolism (20, 53, 175, 176). Here, we review lncRNAs as ceRNAs to modulate the processes of cancer metabolism in PC.

## LINC00261/miR-222-3p/HIPK2

LncRNA LINC00261, firstly identified in hepatocellular carcinoma cells 9 years ago, is located on the 20th chromosome from site 22,560,552 to 22,578,642 (177). An increasing number of studies have indicated that LINC00261 is widely lowly expressed in a variety of cancers and acts as a tumor suppressor contributing to modulating cell proliferation, apoptosis, invasiveness, migration, chemoresistance, angiogenesis, and tumorigenesis *via* multiple molecular mechanisms (177). LINC00261 also plays vital roles in suppression of PC progression by acting as a ceRNA. Zhai et al. (59) demonstrated that overexpression of LINC00261 suppressed PC cell glycolysis *in vitro* and *in vivo*. They further confirmed that LINC00261 inhibited cell glucose metabolism by binding to miR-222-3p to induce homeodomain interacting protein kinase 2 (HIPK2) overexpression and then inactivated HIPK2-mediated ERK/c-Myc pathway, as well as c-Myc target genes [glucose transporter member 1 (GLUT1), hexokinase-2 (HK2), and L-lactate dehydrogenase A chain (LDHA)]. Functionally, miR-222-3p reversed the LINC00261 overexpression-induced decrease in cell glycolysis, similar to HIPK2 and miR-222-3p. Thus, these results revealed that LINC00261 suppressed glycolysis of PC *via* regulating miR-222-3p/HIPK2 ceRNA axis. Moreover, Zhai et al. (59) also found that LINC00261 could reduce c-Myc expression by sequestering Insulin-like growth factor 2 mRNA-binding protein 1 (IGF2BP1) to induce glycolysis suppression. In addition, Liu et al. (178) indicated that LINC00261 repressed c-Myc transcription by physically interacting and binding with the bromo domain of p300/cap binding protein (CBP), preventing the recruitment of p300/CBP to the promoter region of c-Myc. Furthermore, LINC00261 might interact with miR-23a-3p (179) or regulate the miR-552-5p/FOXO3 axis (180) to suppress the development of PC.

## FEZF1-AS1/miR-107/ZNF312B

LncRNA FEZ finger zinc 1 antisense 1 (FEZF1-AS1), transcribed from the opposite strand of its cognate coding gene zinc finger protein 312B (ZNF312B), is a conserved RNA that is located on chromosome 7q31.32 with a length of 2,653 bp (181, 182). Recent research indicates that FEZF1-AS1 is significantly overexpressed and closely related to patient poor prognosis in a variety of malignancies, including nasopharyngeal carcinoma, hepatocellular carcinoma, cervical cancer, colorectal cancer, multiple myeloma, breast cancer, osteosarcoma, lung cancer, gastric cancer, and PC (181, 182). Li et al. (183) and Ye et al. (184) initially identified that FEZF1-AS1 was upregulated in PC tissues through lncRNA expression profile microarray analysis. Subsequently, they confirmed that FEZF1-AS1 and its sense-cognate ZNF312B were markedly expressed in PC tissues by using quantitative real-time PCR (qRT-PCR) and *in situ* hybridization (ISH) (184). FEZF1-AS1 and ZNF312B expression was positively related to advanced American Joint Committee on Cancer (AJCC) stages, nerve invasion, and patients’ poor survival. And a nomogram, which incorporated the AJCC classification with significant prognostic factors neural invasion, ZNF312B expression, and FEZF1-AS1 expression, illustrated that FEZF1-AS1 and ZNF312B expression had important impacts on patient prognosis. Mechanistically, FEZF1-AS1 could act as an endogenous sponge by sequestering miR-107 and thus abolishing the miRNA-induced repressing effect on the ZNF312B expression. Functional experiments also confirmed that the FEZF1-AS1/miR-107/ZNF312B ceRNA axis played a key role in promoting PC cell proliferation, regulating cell cycle, enhancing migration and invasion, and inhibiting apoptosis. More importantly, the FEZF1-AS1/miR-107/ZNF312B pathway contributed to Warburg effect maintenance by promoting glycolytic process, glucose uptake, and lactate production, which met the demands for continuous energy and nutrients to support PC cell tumorigenesis and progression (184). Therefore, the ceRNA-mediated metabolic features of PC provided attractive therapeutic opportunities for treatments. Meanwhile, Ou et al. (185) demonstrated that FEZF1-AS1 could promote PC cell proliferation and invasion through miR-142/HIF-1α axis under hypoxic conditions and exert its oncogenic effect on PC cells through miR-133a/EGFR axis under normoxic conditions.

## SNHG16/miR-195/SREBP2

LncRNA Small Nucleolar RNA Host Gene 16 (SNHG16), initially identified as an oncogene in neuroblastoma, is located on chromosome 17q25.1 and contains two splicing variants (186). Recent studies have shown that SNHG16 is upregulated in a variety of human cancers and significantly correlated with advanced pathological stage, lymph node metastasis, and poor prognosis in cancer patients (186, 187). Meanwhile, increasing evidence has suggested that SNHG16 functions as a tumor-promoting lncRNA that is involved in the regulation of numerous biological processes, including cell cycle, proliferation, apoptosis, migration, and invasion through a variety of potential mechanisms (186, 187). Yu et al. (188) found that SNHG16 accelerated the development of PC and promoted lipogenesis *via* directly regulating miR-195/SREBP2 axis. Knockdown of SNHG16 or Sterol regulatory element binding protein-2 (SREBP2) suppressed PC cell proliferation, migration, and invasion, as well as the lipogenesis that was measured by decreasing the expression of fatty acid synthase (FASN), acetyl-CoA carboxylase 1 (ACACA), and stearoyl-CoA desaturase 1 (SCD1). While overexpression of miR-195 showed the same effect in PC cells. They further confirmed that SNHG16 directly sponged miR-195 from SREBP2 to modulate their expression. Meanwhile, miR-195 inhibitor upregulated the expression of SREBP2 and reversed the effects of shSNHG16 on progression and lipogenesis of PC. Thus, these results showed that SNHG16 promoted lipogenesis of PC *via* regulating miR-195/SREBP2 ceRNA axis. The lncRNA SNHG16/miR-195/SREBP2 axis might be developed as therapeutic targets for treating PC (188). Furthermore, Xu et al. (189) found that SNHG16 contributed to PC cell proliferation, migration, and invasion *via* the miR-302b-3p/SLC2A4 ceRNA axis.

## LncRNAs as CeRNAs Inducing Autophagy

Autophagy is a highly conserved process in response to environmental stresses for ensuring cellular homeostasis through the removal of proteins or dysfunctional organelles (190–192). Existing studies indicate that autophagy plays a dynamic role in cancer initiation, progression, as well as drug resistance, by regulating interactions between the tumor and tumor microenvironment (190–193). There is increasing evidence that a large number of lncRNAs are obviously involved in PC autophagy (20, 53, 191, 194, 195). Identification of the mechanisms by which autophagy is activated in PC will help clarify PC pathogenesis (196). A number of research articles suggested that lncRNAs induce or suppress autophagy through ubiquitin-like modifier-activating enzyme (ATGs), and their signaling pathways may suppress or promote carcinogenesis of PC (192, 194, 195). Here, we describe the recently characterized lncRNAs that function as ceRNAs through inducing or inhibiting autophagy in PC.

## PVT1/miR-20a-5p/ULK1

LncRNA plasmacytoma variant translocation 1 (PVT1), which originated from an intergenic region on chromosome 8, is an important oncogenic lncRNA highly expressed in human malignancies and correlated with patients’ poor prognosis (197, 198). Compared to the majority of lncRNAs, the carcinogenic effect of PVT1 has been confirmed in various tumors. Numerous studies have revealed that PVT1 displays a crucial role to facilitate cancer progression by promoting growth and proliferation, enhancing migration and invasion, suppressing apoptosis, regulating metabolism, maintaining stemness, as well as inducing chemotherapy resistance (197–199). However, current research implies that the mechanisms underlying the carcinogenic role of PVT1 are rather complex. It has been proven that PVT1 can exert its varied oncogenic roles through overexpression and modulation of miRNA expression, protein interactions, targeting of regulatory genes, formation of fusion genes, functioning as a ceRNA, and interactions with myelocytomatosis oncogene (MYC), among many others molecular mechanisms (199, 200). Certainly, identifying the carcinogenic role and molecular mechanism of PVT1 has important implications for therapeutically targeting cancer. Huang et al. (60) demonstrated that PVT1 promoted the development of PC through the PVT1/miR-20a-5p/unc-51-like autophagy-activating kinase 1 (ULK1)/autophagy ceRNA pathway. They found that PVT1 was dramatically upregulated and positively associated with ULK1 protein expression in PC tissues and cells. And overexpression of PVT1 enhanced PC cells autophagy *in vitro* and *in vivo*, whereas knockdown of PVT1 showed the opposite trend. Meanwhile, PVT1 overexpression could promote cell proliferation and colony formation, suppress apoptosis, and increase S phase cells in PC cells; however, the attenuated effects were observed when treated with autophagy inhibitor 3-methyladenine. On the contrary, PVT1 knockdown with treatment of autophagy inducer rapamycin in PC cells would restore proliferation and colony formation, inhibit apoptosis, as well as ascend cell cycle S phase. These data suggested that PVT1 could induce cytoprotective autophagy in PC. Further studies revealed that PVT1 induced autophagy by upregulating ULK1 protein expression. Mechanistically, PVT1 modulated ULK1 expression by sponging miR-20a-5p. Moreover, the expression of PVT1 in high-grade (III + IV) PC tissues was higher than that in low-grade (I + II) tissues. And the overall survival time of patients with high PVT1 expression was significantly shorter than that of patients with low PVT1 expression. Thus, the study demonstrated that PVT1 acted as a sponge to regulate miR-20a-5p and subsequently affected ULK1 expression for inducing autophagy and promoting development of PC (60). Additionally, PVT1 could upregulate the expression of both Pygo2 and ATG14 and thus regulated Wnt/β-catenin signaling and autophagic activity to overcome gemcitabine resistance through sponging miR-619-5p in PC (201). And the ceRNA axes PVT1/miR-448/SERBP1 (202), PVT1/miR-519d-3p/HIF-1α (203), and PVT1/miR-143/HIF-1α (204) might also be potential biomarkers and therapeutic targets for PC.

## LINC01207/miR-143-5p/AGR2

LncRNA LINC01207, located in the 4q32 genomic locus with three exons and two introns, has been reported to be upregulated in multiple types of cancer and associated with the prognosis of patients with poor survival (205–209). Recent studies have demonstrated that LINC01207 performs as an oncogenic lncRNA to promote cell growth, migration, invasion, and enhance apoptosis, as well as maintain stemness *via* ceRNA regulatory mechanism. Liu et al. (210) revealed that silencing of LINC01207 suppressed anterior gradient 2 (AGR2) expressions to facilitate autophagy and apoptosis of PC cells by sponging miR-143-5p. They first confirmed that LINC01207 and AGR2 were highly expressed, while miR-143-5p was poorly expressed in PC tissues when compared to the adjacent tissues. Further studies showed that LINC01207 could directly bind to miR-143-5p, and AGR2 was a target gene of miR-143-5p. And knockdown of LINC01207 could decrease the expression of AGR2 by upregulating miR-143-5p, which indicated that LINC01207 functioned as a ceRNA to upregulate AGR2 expression by sponging miR-143-5p. Moreover, LINC01207 knockdown and miR-143-5p overexpression could inhibit PC cell proliferation, promote apoptosis, and induce autophagy by upregulating the expression of LC3II and beclin-1, while decreasing P62, AGR2, and the ratio of Bcl-2/Bax expression. Thus, LINC01207 silencing inhibited PC progression by inhibiting the mR-143-5p/AGR2 axis, providing a potential target for PC treatment (210).

## ANRIL/miR-181a/HMGB1

LncRNA antisense noncoding RNA in the INK4 locus (ANRIL), initially identified in a kindred of familial melanoma-neural system tumor with a germ-line deletion of the entire CDKN2A/B locus in 2007, is located at the 9p21 region with 3.9 kb length and also named CDKN2B antisense RNA 1 (CDKN2B-AS) (211, 212). It has been proven that ANRIL is implicated in several malignant tumors, and high expression of ANRIL is associated with aggressive clinicopathologic features, such as high histological grade tumor size, advanced tumor–node–metastasis stage, and poor overall survival with the disease (211–213). Additionally, ANRIL participates in tumorigenesis by promoting cell proliferation, migration, invasion, and EMT but inhibiting cell apoptosis through a number of mechanisms (213, 214). Recent studies also show that ANRIL can act as an oncogenic ceRNA to facilitate tumor progression *via* miRNA regulation, including mechanisms involving let-7a (215) and miR-125a (216) in nasopharyngeal carcinoma, miR-99a (217) and miR-449a (218) in gastric cancer, miR-34a (219) in glioma, miR-122-5p (220), miR-191 (221), miR-144 (222), and miR-199a-5p (223) in hepatocellular carcinoma, miR-186 (224) in cervical cancer, let-7a in prostate cancer (225) and colorectal cancer (226), miR-125a-5p (227) in endometrial carcinoma, and miR-199a (228) in breast cancer. In PC, previous research demonstrated that ANRIL was overexpressed in cancer precursors known as intraductal papillary mucinous neoplasms (IPMNs) (229), and ANRIL could promote PC cell migration and invasion through modulation of EMT by activating ATM–E2F1 signaling pathway *in vivo* and *in vitro* (230). Whereas Wang et al. (231) have recently revealed that ANRIL aggravated PC cell gemcitabine chemoresistance by targeting inhibition of miR-181a and activating high-mobility group box-1 (HMGB1)-induced autophagy. They first demonstrated that ANRIL and HMGB1 were obviously higher in PC tissues and cell lines, while miR-181a was significantly lower in both PC tissues and cell lines. And knockdown of ANRIL could inhibit PC cell proliferation, invasion, and migration, as well as the expression of cell adhesion-related proteins. However, downregulation of miR-181a would reverse the inhibitory role of ANRIL knockdown on PC cell, which suggested that the oncogenic role of ANRIL on PC cells might be mediated by miR-181a. Meanwhile, ANRIL knockdown or miR-181a overexpression promoted the expression of LC3 II and Beclin1, while miR-181a inhibition could reverse the inhibition of autophagy by ANRIL knockdown, which indicated that ANRIL-modulated autophagy was mediated by miR-181a. Further studies revealed that miR-181a targeted HMGB1 to suppress PC cell proliferation, invasion, and migration, as well as stimulate autophagy. Mechanistically, ANRIL functioned as a ceRNA to regulate the expression of HMGB1 by inhibiting the activity of miR-181a in PC cells. And ANRIL could enhance PC cells to gemcitabine resistance *via* miR-181a/HMGB1 pathway, which provided new insights and potential targets for the therapy of PC. Moreover, the ANRIL/miR-181a axis also played important roles in laryngeal squamous cell carcinoma, colon cancer, and gastric cancer (232–234).

## LncRNAs as CeRNAs Facilitating Chemoresistance

Chemotherapy resistance causes PC recurrence and failed clinical outcome (235). Cancers can exhibit either intrinsic or acquired chemoresistance to prevent the success of drug treatment (27, 236). It is clear that many factors and signaling pathways are involved in chemoresistance of PC, such as drug transport, metabolism, tumor microenvironment, EMT, DNA damage repair, mutation of drug targets, autophagy, epigenetics. and cancer stem cells (27, 237, 238). However, the molecular mechanisms of chemoresistance remain poorly understood, and the exploration of such mechanisms will help improve the current treatment of PC (238, 239). Since studies have indicated that lncRNAs play critical roles in initiation and progression of PC (20, 27, 53, 237, 240), it is increasingly speculated that the function and mechanism of lncRNA-mediated ceRNA network for chemoresistance regulation.

## GSTM3TV2/let-7/LAT2, OLR1

LncRNA *Homo sapiens* glutathione S-transferase mu 3, transcript variant 2 and noncoding RNA (GSTM3TV2), a novel long intergenic ncRNA encoded from chromosome 1p13.3, has been recently identified as an oncogenic lncRNA to promote gemcitabine resistance through GSTM3TV2/let-7/L-type amino acid transporter 2 (LAT2), oxidized low-density lipoprotein receptor 1 (OLR1) ceRNA pathway in PC (63). The data showed that GSTM3TV2 expression was upregulated in PC tissues and gemcitabine-resistant cell lines and was positively associated with poorer survival in patients with PC. Function studies demonstrated that overexpression of GSTM3TV2 significantly decreased gemcitabine-induced cytotoxicity *in vivo* and *in vitro*, whereas its knockdown reversed these effects in PC. Furthermore, bioinformatics analysis, luciferase assays, and RNA immunoprecipitation assay revealed that GSTM3TV2 was physically associated with let-7 and functioned as ceRNA for let-7 to promote gemcitabine resistance. And let-7 directly targeted LAT2 and OLR1 and suppressed their expressions. LAT2, a transporter of neutral amino acids, activates mechanistic target of rapamycin (mTOR) kinase, thereby inhibiting apoptotic cell death in PC (241). OLR1 is also known to increase HMGA2 transcription by upregulating c-Myc to promote the metastasis in PC (242). LAT2 and OLR1 were upregulated in gemcitabine-resistant cells, and that inhibiting their expression enhanced the chemosensitivity of PC cells to gemcitabine. Meanwhile, GSTM3TV2-mediated chemoresistance could be depressed by knocking down LAT2 and OLR1. Thus, GSTM3TV2 could upregulate the expression of LAT2, OLR1 through competitively sponging let-7 to enhance gemcitabine resistance of PC, which suggested that GSTM3TV2/let-7/LAT2, OLR1 axis might act as a potential biomarker and therapeutic target for PC (63).

## DYNC2H1-4/miR-145

Linc-DYNC2H1-4, an intergenic ncRNA about 281 nt in length, has been originally discovered in human liver (237, 243). A recent study performed by Gao et al. (243) demonstrated that DYNC2H1-4 acted as a sponge of miR-145 to upregulate the expression of its targets, MMP3, Oct4, Lin28, Nanog, Sox2, and ZEB1, thereby promoting EMT progression and CSC formation, which led to chemoresistance in PC cells. They first found that DYNC2H1-4 was upregulated in PC tissues and BxPC-3 gemcitabine-resistant cell line with acquired gemcitabine resistance. Ectopic expression of DYNC2H1-4 promoted migration and invasion as well as pacreatosphere-forming ability in gemcitabine-sensitive PC cells. Knockdown of DYNC2H1-4 suppressed the acquisition of EMT phenotypes and CSC properties in gemcitabine-resistant cells. Mechanistically, DYNC2H1-4 competed with miR-145 to upregulate its targets’ expression. MiR-145 was established as a tumor suppressor, targeting embryonic transcription factors including Lin28, Nanog, Sox2, and Oct4, and also inhibiting the EMT key regulator, ZEB1 expression. Overexpression of DYNC2H1-4 in parental BxPC-3 cells significantly elevated the Lin28, Nanog, Sox2, Oct4, and ZEB1 expressions, while knockdown of DYNC2H1-4 in BxPC-3 gemcitabine-resistant cells showed the opposite effects. Furthermore, upregulation of these miR-145 targets by DYNC2H1-4 was reverted by miR-145 overexpression. In addition, they also found that MMP3, a nearby gene of DYNC2H1-4, was expressed differentially in accordance with DYNC2H1-4 levels in gemcitabine-sensitive and -resistant cell lines. MiR-145 directly targeted MMP3. Overexpression of miR-145 decreased MMP3 expression in gemcitabine-resistant cell lines, and MMP3 upregulation induced by DYNC2H1-4 was downregulated by miR-145, which indicated that DYNC2H1-4/miR-145/MMP3 ceRNA axis was one of the mechanisms by which DYNC2H1-4 was involved in regulating chemoresistance of PC (243).

## GAS5/miR-221/SOCS3

LncRNA growth arrest-specific transcript 5 (GAS5), which is located on chromosome 1q25 and originally found to accumulate in growth-arrested cells, acts as a decoy hormone response element for the glucocorticoid receptor (GR) (244, 245). It has been shown that GAS5 is downregulated and exerted a tumor-suppressive role in diverse cancers, including gastric cancer, non-small cell lung cancer, ovarian cancer, cervical cancer, gliomas, bladder cancer, renal cell carcinoma, and hepatocellular carcinoma (245, 246). The decreased expression of GAS5 has been correlated with poor tumor differentiation, metastasis to the lymph nodes, advanced pathological stages, adverse overall survival, resistance to chemotherapy, and so on (245, 246). Meanwhile, GAS5 interacts with the pathology of variety cancers by inhibiting cell proliferation, suppressing invasion and metastasis, stimulating apoptosis, as well as the induction of cell cycle arrest (237, 244). Recently, it has been reported that GAS5 is also involved in the therapy resistance of cancer by modulating the expression of various gene targets (237, 244). Previous studies have shown that GAS5 was involved in chemoresistance of PC by serving as a ceRNA for miRNA. Liu et al. (247) demonstrated that GAS5 functioned as a competing endogenous RNA for miR-221 to suppress gemcitabine resistance in PC by regulating the miR-221/SOCS3 pathway. They showed that the expression levels of GAS5 and suppressor of cytokine signalling-3 (SOCS3) were downregulated in both PC tissues and cell lines; however, the expression of miR-221 was increased. Upregulation of GAS5 promoted SOCS3 expression and suppressed cell growth, metastasis, and gemcitabine resistance by inhibiting the EMT and tumor stem cell accumulation both *in vivo* and *in vitro*. Mechanistically, GAS5 directly targeted and suppressed miR-221 expression and enhanced SOCS3 expression. Moreover, SOCS3 could reverse the development of miR-221-mediated EMT and stem cell-like phenotype by inhibiting cell proliferation, migration, and chemotherapy resistance. Thus, these results suggested that GAS5/miR-221/SOCS3 ceRNA axis might be a potential therapeutic strategy in PC (247). In addition, GAS5 could negatively regulate miR-181c-5p expression to antagonize gemcitabine and 5-fluorouracil (5-FU) resistance of PC through inactivation of the Hippo signaling (248).

## LncRNAs as CeRNAs Modulating Angiogenesis

Studies have shown that angiogenesis is of great importance in activating the proliferation, invasion, and metastasis of cancer cells, thus playing a crucial role in the initiation and development of solid tumors, including PC (68, 249–252). Many molecular pathways or angiogenic molecules are directly related to angiogenesis, such as VEGF, fibroblast growth factor (FGF), MMP-9, or the platelet-derived growth factor family. Similarly, accumulating studies have also reported that lncRNAs are associated with angiogenesis of cancers (20, 53, 253). In this section, we discuss the latest reports about lncRNAs as ceRNAs involved in angiogenesis of PC.

## CRNDE/miR-451a/CDKN2D

LncRNA Colorectal neoplasia differentially expressed (CRNDE), originally identified to be specifically associated with colorectal cancer, is located on chromosome 16 and is also highly expressed in other cancers, such as lung cancer, hepatocellular carcinoma, gastric cancer, breast cancer, and glioma (254–256). Meanwhile, increasing evidence suggests that CRNDE can function as a crucial tumor promoter to facilitate the progression of carcinogenesis in various cancers. It has been shown that overexpression of CRNDE promotes cell growth and proliferation, enhances migration and invasion, and modulates metabolism while suppressing apoptosis through multiple molecular regulatory networks (254–256). Zhu et al. (257) found that CRNDE promoted the progression and angiogenesis of PC *via* miR-451a/CDKN2D axis. They found that CRNDE was significantly upregulated in PC tissues as well as PC cell lines. And CRNDE overexpression enhanced the progression and angiogenesis of PC cells *in vitro* and *in vivo*. Further studies showed that CRNDE exerted its oncogenic role by sponging miR-451a to upregulate cyclin-dependent kinase inhibitor 2D (CDKN2D) expression. Furthermore, Pearson analysis showed that the expression of CRNDE and miR-451a was negatively correlated, and the expression of miR-451a and CDKN2D was also negatively correlated, while the expression of CRNDE and CDKN2D was positively correlated in PC tissues. Overexpression of miR-451a or CDKN2D knockdown significantly reversed CRNDE-mediated PC cell proliferation, migration, and angiogenesis. Consequently, the above data demonstrated that CRNDE/miR-451a/CDKN2D ceRNA axis might become a potential therapeutic target for PC treatment (257). In addition, Wang et al. (258) reported that CRNDE sponged miR-384 to promote PC cell proliferation and metastasis through upregulating insulin receptor substrate 1 (IRS1).

## LINC00511/miR-29b-3p/VEGFA

LncRNA LINC00511 is transcribed from chromosome 17q24.3 region and upregulated in different malignancies, such as glioma, ovarian cancer, breast cancer, cervical cancer, lung cancer, hepatocellular carcinoma, gastric cancer, and renal cell cancer (259). It has been proven that aberrantly upregulated LINC00511 in malignant tumors is strongly associated with tumor size, clinical stage, lymph node metastasis, and unsatisfactory prognosis. Meanwhile, growing evidence reveals that LINC00511 can accelerate tumor progression by inhibiting malignant cell apoptosis and promoting tumor cell proliferation, migration, invasion, metastasis, chemotherapy resistance, and so on (259). Moreover, recent studies also displayed that LINC00511 played crucial roles in multiple malignant processes of carcinogenesis by serving as a ceRNA. For example, Lu et al. (260) revealed that LINC00511 acted as a ceRNA, which contributed to breast cancer tumorigenesis and stemness by inducing the miR-185-3p/E2F1/Nanog axis, whereas the LINC00511/miR-150/MMP13 ceRNA axis also promoted breast cancer proliferation, migration, and invasion (261). At the same time, LINC00511 facilitated lung squamous cell carcinoma progression *via* sequestering miR-150-5p and activating TADA1 by ceRNA mechanism (262). Additionally, LINC00511 could enhance glioblastoma tumorigenesis and EMT *via* LINC00511/miR-524-5p/YB1/ZEB1 positive feedback loop (263). In PC, Zhao et al. (264) demonstrated that LINC00511 functioned as a ceRNA to mediate the expression of VEGFA through competition for miR-29b-3p, hence serving as a tumor promoter for proliferation, migration, and angiogenesis. They found that LINC00511 was upregulated in PC samples compared with adjacent non-tumoral samples and significantly associated with lymph node metastasis, early recurrence, and poor patient survival. Knockdown of linc00511 impaired tumor proliferation *in vivo* and *in vitro*, concomitant with induction of cell apoptosis. Further studies showed that knockdown of linc00511 blocked PC cell migration, invasion, and angiogenesis *in vitro*. Mechanistically, LINC00511 promoted PC progression through sponging miR-29b-3p to upregulate VEGFA expression. VEGFA knockdown decreased the effect of LINC00511-mediated cell proliferation, invasion, and angiogenesis. In summary, LINC00511/miR-29b-3p/VEGFA axis played a critical role in the tumorigenesis and angiogenesis of PC. Simultaneously, Wang et al. (265) found that miR-29c-3p/LINC00511 may be utilized to indicate prognosis of PC based on ceRNA hypothesis through bioinformatics analysis.

## LncRNAs as CeRNA in Pancreatic Cancer Diagnosis, Prognosis, and Therapy

Diagnosis of diseases by detecting the differential expression of circulating RNA in plasma or serum has become a new technology in the field of noninvasive diagnostic applications (266). Recent studies have found that miRNA can be detected in human peripheral blood despite the large amount of endogenous ribonuclease in blood of cancer patients (267). In addition, a variety of plasma or serum lncRNAs have been characterized as potential tumor markers in human fluids. Ren et al. (268) found that in plasma of patients with prostate cancer, MALAT1 was significantly overexpressed and could significantly discriminate cancer patients from healthy controls. Plasma AA174084 levels were associated with invasion and lymphatic metastasis of gastric cancer and were found to drop markedly on day 15 after the patients received surgery (269). As reported, the aberrant expressions of other lncRNAs have potential to serve as diagnostic or prognostic biomarkers for the human colon, breast, liver, and lung malignancies (270–273). In this section, we discuss some ceRNA networks involved in the diagnosis, prognosis, and therapy of PC.

## UCA1/miR-96-5p/AMOTL2, ERK1, ERK2

LncRNA UCA1 was found to be highly expressed in exosomes derived from hypoxic PC cells and could be transferred to human umbilical vein endothelial cells through the exosomes (168). Further detections revealed the elevated expression levels of UCA1 in exosomes derived from serum of PC patients compared with healthy controls, which was associated with poor survival of PC patients. In addition, UCA1 could promote tumor growth and angiogenesis through the UCA1/miR-96-5p/AMOTL2, ERK1, ERK2 axis.

## PVT1/miR-20b/CCND1

By searching The Cancer Genome Atlas (TCGA) and Genotype-Tissue Expression (GTEx) databases and performing functional enrichment analysis, Zu et al. (274) recognized that pathways in cancer was greatly associated with tumor formation and progression. To identify a meaningful ceRNA network, the stepwise prediction and validation from mRNA to lncRNA were applied according to the ceRNA rules. A total of 11 hub genes, four key miRNAs, and two key lncRNAs were found to be key factors in the ceRNA network, and the PVT1/miR-20b/CCND1 axis was identified as a promising pathway-related ceRNA axis in the progression of PC, which could be considered as encouraging a prognostic biomarker and therapeutic target for PC.

## LncRisk-7

Zhou et al. (275) performed a genome-wide analysis to investigate potential lncRNA-mediated ceRNA interplay based on “ceRNA hypothesis” and uncovered seven novel lncRNAs as functional ceRNAs contributing to PC. Next, based on the expression data and the support vector machine (SVM) algorithm, a seven-lncRNA signature (termed LncRisk-7, including SH3BP5-AS1, STARD4-AS1, ARNTL2-AS1, AC002550.5, RP11-206L10.5, AC016738.4, and RP5-901A4.1) was developed as a novel diagnostic tool, which could significantly improve the early diagnosis of PC. The LncRisk-7 showed promising efficiency in distinguishing PC samples from non-malignant pancreas samples in the training cohort, and its high performance was further confirmed in two independent validation cohorts. Results of the functional experiments demonstrated that the seven lncRNA biomarkers were involved in the regulation of cell cycle, cell death, and cell adhesion of PC cells, mechanistically acting as ceRNAs. Results of this work improved our understanding of the lncRNA-mediated ceRNA regulatory mechanisms in the pathogenesis of PC and provided the LncRisk-7 as potential diagnostic biomarkers.

## A ceRNA Module Comprising of 29 Genes

Using the paired genome-wide expression profiles of lncRNA, miRNA, mRNA, and relationships between them, Zhao et al. (276) constructed a PC-specific hallmark gene-related ceRNA network (HceNet). The characteristics of HceNet was analyzed based on “ceRNA hypothesis,” and a ceRNA module comprising of 12 lncRNAs, 2 miRNAs, and 15 mRNAs was identified as potential prognostic biomarkers associated with the overall survival of PC patients. The prognostic value of ceRNA module biomarkers was further validated to be statistically significant in all the training, the validation, and the entire cohorts. This study provided potential prognostic biomarkers for PC and provided novel insight into the ceRNA-related regulatory mechanism in PC progression.

## A Three-LncRNA Signature

To identify the specific lncRNAs and further analyze their function relating to PC, Shi et al. (277) constructed a global triple network based on the ceRNA theory and RNA-seq data from The Cancer Genome Atlas. Six lncRNAs in the lncRNA–miRNA–mRNA co-expression network were significantly associated with overall survival of PC patients, and a three-lncRNA (LINC00460, C9orf139, and MIR600HG) signature succeeded to predict survival of patients with PC. Protein–protein interaction network data uncovered the association of five genes with the overall survival of PC patients. The findings of this study deepened our understanding in the function of an lncRNA-associated ceRNA network involved in PC pathogenesis and identified the potential prognostic roles of the three-lncRNA signature in PC.

## NAMPTP1/HCG11-hsa-miR-26b-5p-COL12A Subnetwork

By analyzing the expression and survival data of the aberrantly expressed genes in PC according to the systematic mRNA–miRNA–lncRNA/pseudogene network, Jing et al. (278) elucidated the new NAMPTP1/HCG11-hsa-miR-26b-5p-COL12A subnetwork in PC progression. Further validation indicated that the subnetwork might be a candidate diagnostic biomarker or potential therapeutic target for PC.

## An lncRNA–miRNA–mRNA Co-Expression Network

To identify new prognostic markers and develop a multi-mRNAs-based classifier for survival prediction in patients with PC, Weng et al. (29) established an lncRNA–miRNA–mRNA co-expression network that consisted of 66 genes (60 lncRNAs, 3 miRNAs, and 3 mRNAs) relating to the prognosis of PC patients. In addition, a 14-mRNAs-based classifier was constructed based on a training dataset consisting of 178 PC patients. The area under the receiver operating characteristic (AUC) curves in the training dataset for prediction of 1-, 3-, and 5-year OS were 0.719, 0.806, and 0.794, respectively. In the independent validation dataset, the AUC of classifier was 0.604, 0.639, and 0.607, respectively, which showed the good prediction function of the network. The network was associated with PC pathogenesis and might be used as a reference for future molecular biology research.

## Conclusions

The ceRNA interplay is a universal posttranscriptional regulation involving miRNAs and various coding and noncoding RNAs through the functional interactions among them. Comprehensive investigations and understanding into the ceRNA network will greatly increase our knowledge in the underlying molecular mechanisms of cancer pathogenesis. As discussed in this review, the lncRNAs harboring the MREs can specifically sequester miRNAs and function as molecular decoys or sponges, competitively inhibiting the translation and function of their downstream target genes. The lncRNA–miRNA–mRNA ceRNA networks play important regulatory functions in PC progression, including almost all crucial biological processes. As important members of the ceRNA networks, lncRNAs are widely involved in the occurrence of PC, which suggests that plasma lncRNA can to be used as a novel and effective diagnostic biomarker. At the same time, lncRNAs have been found to be involved in the development of the advanced stages of PC, indicating the great potential of these lncRNAs as prognostic biomarkers. More importantly, overexpression or knockdown of related members in the ceRNA networks that are closely associated with the development of PC can significantly inhibit the malignant biological behavior of PC, which suggests them as candidate therapeutic targets for PC.

In recent decades, more and more studies have focused on in-depth explanations of the molecular mechanisms behind the malignant biological behavior of PC. However, the diagnosis and treatment measures related to PC are still limited, and the prognosis of PC has not been significantly improved. At present, the research and understanding of the novel lncRNA-related ceRNA networks are still in the early stage, and the exact mechanisms of their involvement in cancer progression remain largely unknown, which requires in-depth exploration in the molecular mechanisms to provide new advances in the treatment of PC.

## Author Contributions

GX and SP were responsible for gathering information of the related research and designing the review. JJ, XW, RH, FP, XL, and MW were responsible for language editing. JZ, FZ, and RQ have contributed to information interpretation and editing and critical revision of the article. All authors contributed to the article and approved the submitted version.

## Funding

This study was supported by grants from the National Natural Science Foundation of China (No. 81902499, No. 81874205, and No. 81772950).

## Conflict of Interest

The authors declare that the research was conducted in the absence of any commercial or financial relationships that could be construed as a potential conflict of interest.

## Publisher’s Note

All claims expressed in this article are solely those of the authors and do not necessarily represent those of their affiliated organizations, or those of the publisher, the editors and the reviewers. Any product that may be evaluated in this article, or claim that may be made by its manufacturer, is not guaranteed or endorsed by the publisher.

## Glossary

**Table:**

AFAP1-AS1	Actin filament-associated protein 1 antisense RNA 1
AGR2	Anterior gradient 2
ANRIL	Antisense noncoding RNA in the INK4 locus
CDKN2D	Cyclin-dependent kinase inhibitor 2D
ceRNAs	Competing endogenous RNAs
CRNDE	Colorectal neoplasia differentially expressed
CSC	Cancer stem cells
DUSP1	Dual-specificity protein phosphatase 1
EMT	Epithelial–mesenchymal transition
EZH2	Enhancer of zeste homolog 2
FEZF1-AS1	FEZ finger zinc 1 antisense 1
GAS5	Growth arrest−specific transcript 5
GSTM3TV2	Homo sapiens glutathione S-transferase mu 3, transcript variant 2 and noncoding RNA
HIF1A	Hypoxia-Inducible Factor 1A
HIPK2	Homeodomain-interacting protein kinase 2
HMGB1	High-mobility group box-1
HOTAIR	HOX transcript antisense RNA
HULC	Highly upregulated in liver cancer
iASPP	Inhibitor for the apoptosis-stimulating protein of p53
LAT2	L-type amino acid transporter 2
LncRNAs	Long noncoding RNAs
MALAT1	Metastasis-associated lung adenocarcinoma transcript 1
MAPK	Mitogen-activated protein kinase
MIR31HG	MiR-31 host gene
miRNA	MicroRNAs
MREs	MiRNA recognition elements
NORAD	noncoding RNA activated by DNA damage
OLR1	Oxidized low-density lipoprotein receptor 1
OTUD7B	Cezanne
PC	Pancreatic cancer
PRC2	Polycomb repressive complex 2
PVT1	Plasmacytoma variant translocation 1
ROR	Regulator of reprogramming
SNHG16	Small Nucleolar RNA Host Gene 16
SOCS3	Suppressor of cytokine signaling-3
SOX2OT	SOX2 overlapping transcript
SREBP2	Sterol regulatory element-binding protein-2
TGFBR1	Transforming growth factor beta 1
TGFBR2	Transforming growth factor beta 2
THAP9-AS1	THAP9 antisense RNA 1
TUG1	Taurine upregulated gene 1
UCA1	Urothelial cancer-associated 1
ULK1	Unc-51-like autophagy-activating kinase 1
XIST	X inactivation-specific transcript
YAP	Yes-associated protein
