# Supplementary material for: Long Noncoding Competing Endogenous RNA Networks in Pancreatic Cancer
Source: Front Oncol. 2021 Oct 25;11:765216. doi: 10.3389/fonc.2021.765216 (PMC8573238; doi:10.3389/fonc.2021.765216)
Supplement: Supplementary file 1 [file Table_1.docx]

Supplementary Table 1: LncRNA/miRNA/mRNA ceRNA network in PC

| LncRNA | Target miRNA | mRNA | ceRNA role | Biological functions | References |
| --- | --- | --- | --- | --- | --- |
| ABHD11-AS1 | miR-1231 | Cyclin E1 | oncogene | proliferation | (1) |
| ADPGK-AS1 | miR-205-5p | ZEB1 | oncogene | proliferation, migration, invasion, apoptosis | (2) |
| AFAP1-AS1 | miR-384 | ACVR1 | oncogene | stemness | (3) |
| AFAP1-AS1 | miR-146b-5p | EGFR | oncogene | proliferation | (4) |
| AFAP1-AS1 | miR-133a | IGF1R | oncogene | proliferation, invasion, migration | (5) |
| ANRIL | miR-181a | HMGB1 | oncogene | proliferation, invasion, migration, chemoresistance, autophagy | (6) |
| BANCR | miR-195-5p |  | oncogene | proliferation, invasion, migration | (7) |
| C9orf139 | miR-663a | Sox12 | oncogene | proliferation | (8) |
| CASC19 | miR-148b | E2F7 | oncogene | proliferation, migration, invasion | (9) |
| CASC2 | miR‑21 | PTEN | tumor suppressor | migration, invasion | (10) |
| CASC2 | miR‑24 | MUC6 | tumor suppressor | proliferation, migration, invasion, apoptosis | (11) |
| CASC8 | miR-129-5p | TOB1 |  |  | (12) |
| CCAT1 | miR-138-5p | HMGA1 | oncogene | proliferation, apoptosis, angiogenesis | (13) |
| CERS6-AS1 | miR-217 | YWHAG | oncogene | proliferation, metastasis | (14) |
| CERS6-AS1 | miR-15a-5p | FGFR1 | oncogene | proliferation, migration, invasion, apoptosis | (15) |
| CERS6-AS1 | miR-15a-5p, miR-6838-5p | HMGA1 | oncogene | proliferation, migration | (16) |
| CRNDE | miR-384 | IRS1 | oncogene | proliferation, migration, invasion | (17) |
| CRNDE | miR-451a | CDKN2D | oncogene | proliferation, angiogenesis | (18) |
| CYTOR | miR-205-5p | CDK6 | oncogene | proliferation, migration | (19) |
| DANCR | miR-33a-5p | AXL | oncogene | migration, invasion, proliferation | (20) |
| DANCR | miR-135a | NLRP37 | oncogene | proliferation, invasion | (21) |
| DANCR | miR-33b | MMP16 | oncogene | proliferation, migration, invasion | (22) |
| DANCR | miR-214-5p | E2F2 | oncogene | proliferation, metastasis | (23) |
| DAPK1 | miR-182 |  | tumor suppressor | invasion, migration | (24) |
| DGCR5 | miR-3163 | TOP2A |  | proliferation, migration, invasion, apoptosis,  cell cycle, chemoresistance | (25) |
| DGCR5 | miR-27a-3p | BNIP3 | tumor suppressor | apoptosis | (26) |
| DGCR5 | miR-320a | PDCD4 | tumor suppressor | chemoresistance, proliferation, migration, invasion | (27) |
| DIO3OS | miR-122 | ALDOA | oncogene | proliferation, invasion | (28) |
| DLEU1 | miR-381 | CXCR4 | oncogene | proliferation, migration, and invasion | (29) |
| DLEU2 | miR-455 | SMAD2 | oncogene | proliferation, invasion, | (30) |
| DLEU2L | miR-210-3p | BRCA2 | tumor suppressor | chemoresistance, proliferation, migration, invasion, apoptosis | (31) |
| DLX6-AS1 | miR-497-5p | FZD4, FZD6 | oncogene | proliferation, migration, invasion | (32) |
| DLX6‑AS1 | miR-181b | ZEB2 | oncogene | proliferation, invasion, EMT | (33) |
| DNAH17-AS1 | miR-432-5p | PPME1 | oncogene | apoptosis, proliferation, invasion, migration | (34) |
| DUXAP8 | miR-448 | WTAP | oncogene | migration, invasion, proliferation | (35) |
| DYNC2H1-4 | miR-145 | ZEB1, Lin28, Nanog, Sox2, Oct4, MMP3 | oncogene | chemoresistance,stemness | (36) |
| FEZF1-AS1 | miR-107 | ZNF312B | oncogene | proliferation, migration, invasion, | (37) |
| FEZF1-AS1 | miR-142 | HIF-1α | oncogene | proliferation, invasion | (38) |
| FEZF1-AS1 | miR-133a | EGFR | oncogene | proliferation, invasion | (38) |
| FGD5-AS1 | miR-520a-3p | KIAA1522 | oncogene | proliferation, migration | (39) |
| FTX | miR-513b-5p |  | oncogene | invasion, proliferation | (40) |
| GAS5 | miR-221 | SOCS3 | tumor suppressor | chemoresistance, metastasis, proliferation | (41) |
| GAS5 | miR‑32‑5p | PTEN | tumor suppressor | migration, invasion, proliferation, apoptosis | (42) |
| GAS5 | miR-181c-5p |  | tumor suppressor | chemoresistance | (43) |
| GATA3‑AS1 | miR-30b-5p | Tex10 | oncogene | proliferation, invasion, apoptosis, stemness | (44) |
| GSTM3TV2 | let-7 | LAT2, OLR1 | oncogene | chemoresistance | (45) |
| H19 | miR-194 | PFTK1 | oncogene | proliferation, migration | (46) |
| H19 | let-7 | HMGA2 | oncogene | invasion, migration | (47) |
| HCG11 | miR-579-3p | MDM2 | oncogene | proliferation, invasion, migration | (48) |
| HCP5 | miR-214-3p | HDGF | oncogene | chemoresistance, proliferation, invasion, migration, apoptosis, autophagy | (49) |
| HOTAIR | miR-34a |  | oncogene | stemness, invasion, migration | (50) |
| HOTAIR | miR-613 | notch3 | oncogene | proliferation | (51) |
| HOTTIP | miR-497 |  | oncogene | proliferation, invasion | (52) |
| HOTTIP | miR-137 |  | oncogene | chemoresistance, proliferation, apoptosis | (53) |
| HULC | miR-133b |  |  | invasion, migration | (54) |
| ITGB2-AS1 | miR-4319 | RAF1 | oncogene | proliferation, apoptosis, cell cycle, migration, invasion | (55) |
| LINC00052 | miR-330-3p |  | tumor suppressor | proliferation, cell cycle, apoptosis, migration, invasion | (56) |
| LINC00152 | miR-150 |  | oncogene | proliferation, migration, invasion | (57) |
| LINC00261 | miR-222-3p | HIPK2 | tumor suppressor | glycolysis, proliferation, cell cycle, apoptosis | (58) |
| LINC00261 | miR-23a-3p |  | tumor suppressor | proliferation, invasion, apoptosis | (59) |
| LINC00339 | miR-497-5p | IGF1R | oncogene | proliferation, invasion, migration | (60) |
| LINC00346 | miR-188-3p | BRD4 | oncogene | chemoresistance, proliferation, cell cycle | (61) |
| LINC00460 | miR-491-5p |  | oncogene | proliferation, migration, cell cycle, apoptosis | (62) |
| LINC00460 | miR-320b | ARF1 | oncogene | proliferation, migration, invasion, apoptosis | (63) |
| LINC00462 | miR-665 | TGFBR1, TGFBR2 | oncogene | proliferation, apoptosis, migration, invasion, EMT | (64) |
| LINC00473 | miR-195-5p | PD-L1 | oncogene | Immunotherapy, apoptosis, proliferation, invasion, migration | (65) |
| LINC00511 | miR-29b-3p | VEGFA | oncogene | proliferation, invasion, angiogenesis | (66) |
| LINC00514 | miR-28-5p | Rap1b | oncogene | proliferation, migration, invasion | (67) |
| LINC00657 | miR-433 | PAK4 | oncogene | proliferation, apoptosis, migration, invasion | (68) |
| LINC00857 | miR-340-5p | TGFA | oncogene | proliferation, invasion, migration | (69) |
| LINC00857 | miR-150-5p | E2F3 | oncogene | proliferation, apoptosis | (70) |
| LINC00941 | miR-335-5p | ROCK1 | oncogene | proliferation, metastasis, EMT | (71) |
| LINC00941 | miR-873-3p | ATXN2 | oncogene | proliferative, invasion, migration | (72) |
| LINC00958 | miR-330-5p | PAX8 | oncogene | EMT, invasion, metastasis, proliferation | (73) |
| LINC00960 | miR-146a-5p | IRAK1 | oncogene | proliferation, migration, invasion | (74) |
| LINC00976 | miR-137 | OTUD7B | oncogene | proliferation, migration, invasion | (75) |
| LINC00994 | miR-765-3p | RUNX2 | oncogene | proliferation, migration, invasion, cell cycle, apoptosis | (76) |
| LINC01006 | miR‑2682‑5p | HOXB8 | oncogene | proliferation, invasion, migration | (77) |
| LINC01111 | miR-3924 | DUSP1 | tumor suppressor | proliferation, cell cycle, invasion, migration | (78) |
| LINC01207 | miR-143-5p | AGR2 | oncogene | autophagy, apoptosis | (79) |
| LINC01410 | miR-497-5p | IFITM3 | oncogene | proliferation, migration | (80) |
| LINC01420 | miR-494-3p | MYC | oncogene | proliferation, EMT | (81) |
| LINC01448 | miR-505 | HK2 | oncogene | proliferation, invasion, stemness, glycolysis | (82) |
| LINC01559 | miR-607 | YAP | oncogene | proliferation, migration | (83) |
| LINC01559 | miR-1343-3p | RAF1 | oncogene | migration, invasion, proliferation | (84) |
| LINC01963 | miR-641 | TMEFF2 | tumor suppressor | cell cycle, proliferation, invasion, apoptosis | (85) |
| LINP1 | miR-491-3p |  | oncogene | proliferation, metastasis | (86) |
| LNC00673 | miR-504 | HNF1A | tumor suppressor | apoptosis, proliferation, migration | (87) |
| LOXL1-AS1 | miR-28-5p | SEMA7A | oncogene | proliferation, migration | (88) |
| LUCAT1 | miR-539 |  | oncogene | proliferation, cell cycle, migration, invasion | (89) |
| MAFG-AS1 | miR-3196 | NFIX | oncogene | proliferation, migration | (90) |
| MALAT1 | miR-200c-3p | ZEB1 | oncogene | migration, invasion | (91) |
| MALAT1 | miR-217 | KRAS | oncogene | proliferation, migration, invasion | (92) |
| MALAT1 | miR-216a |  | oncogene | cell cycle, chemoresistance | (93) |
| MCM3AP-AS1 | miR-138-5p | FOXK1 | oncogene | proliferation, migration, invasion | (94) |
| MEG3 | miR-374a-5p | PTEN | tumor suppressor | growth, metastasis | (95) |
| MIAT | miR-133 |  | oncogene | proliferation, migration, invasion | (96) |
| MIR155HG | miR-802 |  | oncogene | proliferation, apoptosis | (97) |
| MIR210HG | miR-125b-5p | HK2, PKM2 | oncogene | glycolysis, proliferation, invasion, migration | (98) |
| MIR31HG | miR-193b |  | oncogene | proliferation, apoptosis, cell cycle, invasion | (99) |
| MSC-AS1 | miR-29b-3p | CDK14 | oncogene | proliferation, chemoresistance | (100) |
| NEAT1 | miR-302a-3p | RELA | oncogene | proliferation, migration | (101) |
| NEAT1 | miR-506-3p |  | oncogene | proliferation, cell cycle, apoptosis | (102) |
| NORAD | miR-125a-3p | RhoA | oncogene | EMT, migration, invasion | (103) |
| NUTF2P3-001 | miR-3923 | KRAS | oncogene | proliferation, invasion | (104) |
| OIP5-AS1 | miR-429 | FOXD1 | oncogene | proliferation | (105) |
| OIP5-AS1 | miR-186-5p | NGFR | oncogene | migration, invasion | (106) |
| OIP5-AS1 | miR-342-3p | AGR2 | oncogene | proliferation, cell cycle | (107) |
| OIP5-AS1 | miR-320b | FOXM1 | oncogene | proliferation, migration, invasion, apoptosis | (108) |
| PART1 | miR-122 |  | oncogene | proliferation, invasion, apoptosis | (109) |
| PCAT6 | miR-185-5p | CBX2 | oncogene | proliferation, migration, invasion | (110) |
| PCED1B‑AS1 | miR-411-3p | HIF-1α | oncogene | proliferation, invasion, EMT | (111) |
| PSMB8-AS1 | miR-382-3p | STAT1 | oncogene | proliferation, metastasis | (112) |
| PTTG3P | miR-132/212-3p | FoxM1 | oncogene | proliferation, metastasis | (113) |
| PVT1 | miR-619-5p | Pygo2, ATG14 | oncogene | chemoresistance | (114) |
| PVT1 | miR-20a-5p | ULK1 | oncogene | autophagy, proliferation | (115) |
| PVT1 | miR-448 | SERBP1 | oncogene | proliferation, migration, EMT | (116) |
| PVT1 | miR-519d-3p | HIF-1A | oncogene | proliferation, glycolysis, invasion | (117) |
| PXN-AS1 | miR-3064 | PIP4K2B | tumor suppressor | proliferation, invasion, stemness | (118) |
| ROR | let-7 |  | oncogene | proliferation, migration, invasion, stemness | (119) |
| ROR | miR‑124 | PTBP1, PKM2 | oncogene | chemoresistance | (120) |
| ROR | miR-145 | Nanog | oncogene | stemness, proliferation, apoptosis, migration | (121) |
| SBF2-AS1 | miR-142-3p | TWF1 | oncogene | chemoresistance, proliferation, EMT, apoptosis | (122) |
| SBF2-AS1 | miR-122-5p | XIAP | oncogene | tumor microenvironment | (123) |
| SNHG1 | miR-195 | Cyclin D1 | oncogene | proliferation, cell cycle | (124) |
| SNHG12 | miR-320b |  | oncogene | EMT, proliferation, invasion | (125) |
| SNHG14 | miR-613 | ANXA2 | oncogene | proliferation, invasion, apoptosis | (126) |
| SNHG14 | miR-101 |  | oncogene | chemoresistance, proliferation, migration, invasion | (127) |
| SNHG16 | miR-218-5p | HMGB1 | oncogene | proliferation, migration, invasion | (128) |
| SNHG16 | miR-302b-3p | SLC2A4 | oncogene | proliferation, migration, invasion | (129) |
| SNHG16 | miR-195 | SREBP2 | oncogene | proliferation, migration, invasion | (130) |
| SNHG17 | miR-942 |  | oncogene | proliferation, migration, invasion, apoptosis | (131) |
| SNHG6 | miR-26a-5p | FUBP1 | oncogene | proliferation, migration, invasion | (132) |
| SNHG7 | miR-342-3p | ID4 | oncogene | proliferation, migration, invasion | (133) |
| SNHG7 | miR-146b-5p | Robo1 | oncogene | proliferation, migration, invasion, apoptosis | (134) |
| SOX2OT | miR-200 | Sox2 | oncogene | EMT, stemness, invasion, metastasis | (135) |
| THAP9-AS1 | miR-484 | YAP | oncogene | proliferation | (136) |
| TMPO-AS1 | miR-383-5p | SOX11 | oncogene | proliferation, migration, invasion | (137) |
| TP53TG1 | miR-96 | KRAS | oncogene | proliferation, apoptosis, migration, invasion | (138) |
| TP73-AS1 | miR-128-3p | GOLM1 | oncogene | proliferation, migration, invasion | (139) |
| TP73-AS1 | miR-200a | MMP14 | oncogene | migration, invasion | (140) |
| TP73-AS1 | miR-141 | BDH2 | oncogene | migration, invasion | (141) |
| TUG1 | miR-376b-3p | DPD | oncogene | chemoresistance | (142) |
| TUG1 | miR-29c |  | oncogene | proliferation, invasion | (143) |
| TUG1 | miR-382 | EZH2 | oncogene | proliferation, migration, EMT | (144) |
| TUSC7 | miR-371a-5p |  | tumor suppressor | proliferation, apoptosis, migration, invasion, EMT, stemness | (145) |
| UCA1 | miR-96-5p | AMOTL2 | oncogene | angiogenesis, proliferation | (146) |
| UCA1 | miR-590-3p | KRAS | oncogene | stemness, proliferation | (147) |
| UCA1 | miR-107 | ITGA2 | oncogene | migration, invasion | (148) |
| UCA1 | miR-135a |  | oncogene | proliferation, apoptosis, migration, invasion | (149) |
| UCA1 | miR-96 | FOXO3 | oncogene | proliferation, cell cycle, apoptosis, invasion, migration | (150) |
| XIST | miR-429 | ZEB1 | oncogene | migration, invasion, EMT | (151) |
| XIST | miR-133a | EGFR | oncogene | proliferation | (152) |
| XIST | miR-34a-5p |  | oncogene | proliferation, migration, invasion, apoptosis | (153) |
| XIST | miR-34a | YAP | oncogene | EMT, proliferation, invasion, apoptosis | (154) |
| XIST | miR-141-3p | TGF-β2 | oncogene | migration, invasion, proliferation | (155) |
| XIST | miR-140, miR-124 | iASPP | oncogene | proliferation, cell cycle | (156) |
| XIST | miR-137 | Notch1 | oncogene | proliferation | (157) |
| ZEB1-AS1 | miR-505 | TRIB2 | oncogene | proliferation, migration, invasion | (158) |
| ZEB2-AS1 | miR-204 | HMGB1 | oncogene | proliferation, migration | (159) |
| ZFAS1 | miR-3924 | ROCK2 | oncogene | metastasis | (160) |

lncRNA: long noncoding RNA; ceRNA: competitive endogenous RNA; miRNA: microRNA; mRNA: messenger RNA; PC: pancreatic cancer; EMT: epithelial-mesenchymal transition.

Reference

1. Liu B, Wang W, Sun S, Ding H, Lan L, Li X, et al. Knockdown of lncRNA ABHD11-AS1 Suppresses the Tumorigenesis of Pancreatic Cancer via Sponging miR-1231. OncoTargets and therapy. 2020;13:11347-11358. eng. Epub 2020/11/13. doi:10.2147/ott.S259598.

2. Song S, Yu W, Lin S, Zhang M, Wang T, Guo S, et al. LncRNA ADPGK-AS1 promotes pancreatic cancer progression through activating ZEB1-mediated epithelial-mesenchymal transition. Cancer biology & therapy. 2018 Jul 3;19(7):573-583. eng. Epub 2018/04/19. doi:10.1080/15384047.2018.1423912.

3. Wu XB, Feng X, Chang QM, Zhang CW, Wang ZF, Liu J, et al. Cross-talk among AFAP1-AS1, ACVR1 and microRNA-384 regulates the stemness of pancreatic cancer cells and tumorigenicity in nude mice. Journal of experimental & clinical cancer research : CR. 2019 Feb 28;38(1):107. eng. Epub 2019/03/02. doi:10.1186/s13046-019-1051-0.

4. Zhou J, Liu M, Chen Y, Xu S, Guo Y, Zhao L. Cucurbitacin B suppresses proliferation of pancreatic cancer cells by ceRNA: Effect of miR-146b-5p and lncRNA-AFAP1-AS1. Journal of cellular physiology. 2019 Apr;234(4):4655-4667. eng. Epub 2018/09/13. doi:10.1002/jcp.27264.

5. Chen B, Li Q, Zhou Y, Wang X, Zhang Q, Wang Y, et al. The long coding RNA AFAP1-AS1 promotes tumor cell growth and invasion in pancreatic cancer through upregulating the IGF1R oncogene via sequestration of miR-133a. Cell Cycle. 2018;17(16):1949-1966. eng. Epub 2018/10/10. doi:10.1080/15384101.2018.1496741.

6. Wang L, Bi R, Li L, Zhou K, Yin H. lncRNA ANRIL aggravates the chemoresistance of pancreatic cancer cells to gemcitabine by targeting inhibition of miR-181a and targeting HMGB1-induced autophagy. Aging. 2021 Aug 10;13(15):19272-19281. eng. Epub 2021/08/11. doi:10.18632/aging.203251.

7. Wu X, Xia T, Cao M, Zhang P, Shi G, Chen L, et al. LncRNA BANCR Promotes Pancreatic Cancer Tumorigenesis via Modulating MiR-195-5p/Wnt/β-Catenin Signaling Pathway. Technology in cancer research & treatment. 2019 Jan-Dec;18:1533033819887962. eng. Epub 2019/11/27. doi:10.1177/1533033819887962.

8. Ge JN, Yan D, Ge CL, Wei MJ. LncRNA C9orf139 can regulate the growth of pancreatic cancer by mediating the miR-663a/Sox12 axis. World journal of gastrointestinal oncology. 2020 Nov 15;12(11):1272-1287. eng. Epub 2020/12/01. doi:10.4251/wjgo.v12.i11.1272.

9. Lu T, Wei GH, Wang J, Shen J. LncRNA CASC19 contributed to the progression of pancreatic cancer through modulating miR-148b/E2F7 axis. European review for medical and pharmacological sciences. 2020 Oct;24(20):10462-10471. eng. Epub 2020/11/07. doi:10.26355/eurrev_202010_23399.

10. Zhang H, Feng X, Zhang M, Liu A, Tian L, Bo W, et al. Long non-coding RNA CASC2 upregulates PTEN to suppress pancreatic carcinoma cell metastasis by downregulating miR-21. Cancer Cell Int. 2019;19:18. eng. Epub 2019/01/25. doi:10.1186/s12935-019-0728-y.

11. Xu DF, Wang LS, Zhou JH. Long non‑coding RNA CASC2 suppresses pancreatic cancer cell growth and progression by regulating the miR‑24/MUC6 axis. International journal of oncology. 2020 Feb;56(2):494-507. eng. Epub 2020/01/03. doi:10.3892/ijo.2019.4937.

12. Zhu W, Gao W, Deng Y, Yu X, Zhu H. Identification and Development of Long Non-coding RNA Associated Regulatory Network in Pancreatic Adenocarcinoma. OncoTargets and therapy. 2020;13:12083-12096. eng. Epub 2020/12/03. doi:10.2147/ott.S265036.

13. Han W, Sulidankazha Q, Nie X, Yilidan R, Len K. Pancreatic cancer cells-derived exosomal long non-coding RNA CCAT1/microRNA-138-5p/HMGA1 axis promotes tumor angiogenesis. Life sciences. 2021 Aug 1;278:119495. eng. Epub 2021/04/20. doi:10.1016/j.lfs.2021.119495.

14. Xu J, Wang J, He Z, Chen P, Jiang X, Chen Y, et al. LncRNA CERS6-AS1 promotes proliferation and metastasis through the upregulation of YWHAG and activation of ERK signaling in pancreatic cancer. Cell Death Dis. 2021 Jun 24;12(7):648. eng. Epub 2021/06/26. doi:10.1038/s41419-021-03921-3.

15. Yun Z, Meng F, Li S, Zhang P. Long non-coding RNA CERS6-AS1 facilitates the oncogenicity of pancreatic ductal adenocarcinoma by regulating the microRNA-15a-5p/FGFR1 axis. Aging. 2021 Feb 13;13(4):6041-6054. eng. Epub 2021/02/14. doi:10.18632/aging.202540.

16. Shen R, Wang X, Wang S, Zhu D, Li M. Long Noncoding RNA CERS6-AS1 Accelerates the Proliferation and Migration of Pancreatic Cancer Cells by Sequestering MicroRNA-15a-5p and MicroRNA-6838-5p and Modulating HMGA1. Pancreas. 2021 Apr 1;50(4):617-624. eng. Epub 2021/05/04. doi:10.1097/mpa.0000000000001806.

17. Wang G, Pan J, Zhang L, Wei Y, Wang C. Long non-coding RNA CRNDE sponges miR-384 to promote proliferation and metastasis of pancreatic cancer cells through upregulating IRS1. Cell Prolif. 2017 Dec;50(6). eng. Epub 2017/09/25. doi:10.1111/cpr.12389.

18. Zhu HY, Gao YJ, Wang Y, Liang C, Zhang ZX, Chen Y. LncRNA CRNDE promotes the progression and angiogenesis of pancreatic cancer via miR-451a/CDKN2D axis. Translational oncology. 2021 Jul;14(7):101088. eng. Epub 2021/04/22. doi:10.1016/j.tranon.2021.101088.

19. Zhu H, Shan Y, Ge K, Lu J, Kong W, Jia C. LncRNA CYTOR promotes pancreatic cancer cell proliferation and migration by sponging miR-205-5p. Pancreatology : official journal of the International Association of Pancreatology (IAP) [et al]. 2020 Sep;20(6):1139-1148. eng. Epub 2020/08/01. doi:10.1016/j.pan.2020.05.004.

20. Chen L, Liu J, Tang T, Zhang YC, Liu MZ, Xu LY, et al. lncRNA differentiation antagonizing nonprotein coding RNA overexpression accelerates progression and indicates poor prognosis in pancreatic ductal adenocarcinoma. OncoTargets and therapy. 2018;11:7955-7965. eng. Epub 2018/12/07. doi:10.2147/ott.S167065.

21. Tang Y, Cao G, Zhao G, Wang C, Qin Q. LncRNA differentiation antagonizing non-protein coding RNA promotes proliferation and invasion through regulating miR-135a/NLRP37 axis in pancreatic cancer. Invest New Drugs. 2020 Jun;38(3):714-721. eng. Epub 2019/07/04. doi:10.1007/s10637-019-00798-0.

22. Luo Y, Wang Q, Teng L, Zhang J, Song J, Bo W, et al. LncRNA DANCR promotes proliferation and metastasis in pancreatic cancer by regulating miRNA-33b. FEBS open bio. 2020 Jan;10(1):18-27. eng. Epub 2019/09/14. doi:10.1002/2211-5463.12732.

23. Yao Z, Chen Q, Ni Z, Zhou L, Wang Y, Yang Y, et al. Long Non-Coding RNA Differentiation Antagonizing Nonprotein Coding RNA (DANCR) Promotes Proliferation and Invasion of Pancreatic Cancer by Sponging miR-214-5p to Regulate E2F2 Expression. Medical science monitor : international medical journal of experimental and clinical research. 2019 Jun 19;25:4544-4552. eng. Epub 2019/06/20. doi:10.12659/msm.916960.

24. Xu X, Wang X, Geng C, Nie X, Bai C. Long-chain non-coding RNA DAPK1 targeting miR-182 regulates pancreatic cancer invasion and metastasis through ROCK-1/rhoa signaling pathway. Int J Clin Exp Pathol. 2017;10(9):9273-9283. eng. Epub 2017/09/01.

25. Liu SL, Cai C, Yang ZY, Wu ZY, Wu XS, Wang XF, et al. DGCR5 is activated by PAX5 and promotes pancreatic cancer via targeting miR-3163/TOP2A and activating Wnt/β-catenin pathway. Int J Biol Sci. 2021;17(2):498-513. eng. Epub 2021/02/23. doi:10.7150/ijbs.55636.

26. Li X, Zhou S, Fan T, Feng X. lncRNA DGCR 5/miR‑27a‑3p/BNIP3 promotes cell apoptosis in pancreatic cancer by regulating the p38 MAPK pathway. Int J Mol Med. 2020 Aug;46(2):729-739. eng. Epub 2020/07/07. doi:10.3892/ijmm.2020.4632.

27. Yong S, Yabin Y, Bing Z, Chuanrong Z, Dianhua G, Jianhuai Z, et al. Reciprocal regulation of DGCR5 and miR-320a affects the cellular malignant phenotype and 5-FU response in pancreatic ductal adenocarcinoma. Oncotarget. 2017 Oct 31;8(53):90868-90878. eng. Epub 2017/12/07. doi:10.18632/oncotarget.18377.

28. Cui K, Jin S, Du Y, Yu J, Feng H, Fan Q, et al. Long noncoding RNA DIO3OS interacts with miR-122 to promote proliferation and invasion of pancreatic cancer cells through upregulating ALDOA. Cancer Cell Int. 2019;19:202. eng. Epub 2019/08/07. doi:10.1186/s12935-019-0922-y.

29. Gao S, Cai Y, Zhang H, Hu F, Hou L, Xu Q. Long noncoding RNA DLEU1 aggravates pancreatic ductal adenocarcinoma carcinogenesis via the miR-381/CXCR4 axis. Journal of cellular physiology. 2019 May;234(5):6746-6757. eng. Epub 2018/11/02. doi:10.1002/jcp.27421.

30. Xu B, Gong X, Zi L, Li G, Dong S, Chen X, et al. Silencing of DLEU2 suppresses pancreatic cancer cell proliferation and invasion by upregulating microRNA-455. Cancer science. 2019 May;110(5):1676-1685. eng. Epub 2019/03/07. doi:10.1111/cas.13987.

31. Xu F, Wu H, Xiong J, Peng T. Long Non-coding RNA DLEU2L Targets miR-210-3p to Suppress Gemcitabine Resistance in Pancreatic Cancer Cells via BRCA2 Regulation. Frontiers in molecular biosciences. 2021;8:645365. eng. Epub 2021/05/11. doi:10.3389/fmolb.2021.645365.

32. Yang J, Ye Z, Mei D, Gu H, Zhang J. Long noncoding RNA DLX6-AS1 promotes tumorigenesis by modulating miR-497-5p/FZD4/FZD6/Wnt/β-catenin pathway in pancreatic cancer. Cancer management and research. 2019;11:4209-4221. eng. Epub 2019/05/24. doi:10.2147/cmar.S194453.

33. An Y, Chen XM, Yang Y, Mo F, Jiang Y, Sun DL, et al. LncRNA DLX6-AS1 promoted cancer cell proliferation and invasion by attenuating the endogenous function of miR-181b in pancreatic cancer. Cancer Cell Int. 2018;18:143. eng. Epub 2018/09/27. doi:10.1186/s12935-018-0643-7.

34. Xu T, Lei T, Li SQ, Mai EH, Ding FH, Niu B. DNAH17-AS1 promotes pancreatic carcinoma by increasing PPME1 expression via inhibition of miR-432-5p. World J Gastroenterol. 2020 Apr 21;26(15):1745-1757. eng. Epub 2020/05/01. doi:10.3748/wjg.v26.i15.1745.

35. Li JR, Liu L, Luo H, Chen ZG, Wang JH, Li NF. Long Noncoding RNA DUXAP8 Promotes Pancreatic Carcinoma Cell Migration and Invasion Via Pathway by miR-448/WTAP/Fak Signaling Axis. Pancreas. 2021 Mar 1;50(3):317-326. eng. Epub 2021/02/25. doi:10.1097/mpa.0000000000001751.

36. Gao Y, Zhang Z, Li K, Gong L, Yang Q, Huang X, et al. Linc-DYNC2H1-4 promotes EMT and CSC phenotypes by acting as a sponge of miR-145 in pancreatic cancer cells. Cell Death Dis. 2017 Jul 13;8(7):e2924. eng. Epub 2017/07/14. doi:10.1038/cddis.2017.311.

37. Ye H, Zhou Q, Zheng S, Li G, Lin Q, Ye L, et al. FEZF1-AS1/miR-107/ZNF312B axis facilitates progression and Warburg effect in pancreatic ductal adenocarcinoma. Cell Death Dis. 2018 Jan 18;9(2):34. eng. Epub 2018/01/20. doi:10.1038/s41419-017-0052-1.

38. Ou ZL, Zhang M, Ji LD, Luo Z, Han T, Lu YB, et al. Long noncoding RNA FEZF1-AS1 predicts poor prognosis and modulates pancreatic cancer cell proliferation and invasion through miR-142/HIF-1α and miR-133a/EGFR upon hypoxia/normoxia. Journal of cellular physiology. 2019 Jan 28. eng. Epub 2019/01/30. doi:10.1002/jcp.28188.

39. Lin J, Liao S, Liu Z, Li E, Wu X, Zeng W. LncRNA FGD5-AS1 accelerates cell proliferation in pancreatic cancer by regulating miR-520a-3p/KIAA1522 axis. Cancer biology & therapy. 2021 Mar 4;22(3):257-266. eng. Epub 2021/04/03. doi:10.1080/15384047.2021.1883184.

40. Li S, Zhang Q, Liu W, Zhao C. Silencing of FTX suppresses pancreatic cancer cell proliferation and invasion by upregulating miR-513b-5p. BMC cancer. 2021 Mar 18;21(1):290. eng. Epub 2021/03/20. doi:10.1186/s12885-021-07975-6.

41. Liu B, Wu S, Ma J, Yan S, Xiao Z, Wan L, et al. lncRNA GAS5 Reverses EMT and Tumor Stem Cell-Mediated Gemcitabine Resistance and Metastasis by Targeting miR-221/SOCS3 in Pancreatic Cancer. Molecular therapy Nucleic acids. 2018 Dec 7;13:472-482. eng. Epub 2018/11/06. doi:10.1016/j.omtn.2018.09.026.

42. Gao ZQ, Wang JF, Chen DH, Ma XS, Wu Y, Tang Z, et al. Long non-coding RNA GAS5 suppresses pancreatic cancer metastasis through modulating miR-32-5p/PTEN axis. Cell & bioscience. 2017;7:66. eng. Epub 2017/12/12. doi:10.1186/s13578-017-0192-0.

43. Gao ZQ, Wang JF, Chen DH, Ma XS, Yang W, Zhe T, et al. Long non-coding RNA GAS5 antagonizes the chemoresistance of pancreatic cancer cells through down-regulation of miR-181c-5p. Biomedicine & pharmacotherapy = Biomedecine & pharmacotherapie. 2018 Jan;97:809-817. eng. Epub 2017/11/08. doi:10.1016/j.biopha.2017.10.157.

44. Liu Y, Xu G, Li L. LncRNA GATA3‑AS1‑miR‑30b‑5p‑Tex10 axis modulates tumorigenesis in pancreatic cancer. Oncology reports. 2021 May;45(5). eng. Epub 2021/03/25. doi:10.3892/or.2021.8010.

45. Xiong G, Liu C, Yang G, Feng M, Xu J, Zhao F, et al. Long noncoding RNA GSTM3TV2 upregulates LAT2 and OLR1 by competitively sponging let-7 to promote gemcitabine resistance in pancreatic cancer. J Hematol Oncol. 2019 Sep 12;12(1):97. eng. Epub 2019/09/14. doi:10.1186/s13045-019-0777-7.

46. Sun Y, Zhu Q, Yang W, Shan Y, Yu Z, Zhang Q, et al. LncRNA H19/miR-194/PFTK1 axis modulates the cell proliferation and migration of pancreatic cancer. J Cell Biochem. 2019 Mar;120(3):3874-3886. eng. Epub 2018/11/27. doi:10.1002/jcb.27669.

47. Ma C, Nong K, Zhu H, Wang W, Huang X, Yuan Z, et al. H19 promotes pancreatic cancer metastasis by derepressing let-7's suppression on its target HMGA2-mediated EMT. Tumour biology : the journal of the International Society for Oncodevelopmental Biology and Medicine. 2014 Sep;35(9):9163-9. eng. Epub 2014/06/13. doi:10.1007/s13277-014-2185-5.

48. Xu J, Xu W, Yang X, Liu Z, Sun Q. LncRNA HCG11/miR-579-3p/MDM2 axis modulates malignant biological properties in pancreatic carcinoma via Notch/Hes1 signaling pathway. Aging. 2021 Jun 21;13(12):16471-16484. eng. Epub 2021/07/08. doi:10.18632/aging.203167.

49. Liu Y, Wang J, Dong L, Xia L, Zhu H, Li Z, et al. Long Noncoding RNA HCP5 Regulates Pancreatic Cancer Gemcitabine (GEM) Resistance By Sponging Hsa-miR-214-3p To Target HDGF. OncoTargets and therapy. 2019;12:8207-8216. eng. Epub 2019/10/22. doi:10.2147/ott.S222703.

50. Deng S, Wang J, Zhang L, Li J, Jin Y. LncRNA HOTAIR Promotes Cancer Stem-Like Cells Properties by Sponging miR-34a to Activate the JAK2/STAT3 Pathway in Pancreatic Ductal Adenocarcinoma. OncoTargets and therapy. 2021;14:1883-1893. eng. Epub 2021/03/20. doi:10.2147/ott.S286666.

51. Cai H, Yao J, An Y, Chen X, Chen W, Wu D, et al. LncRNA HOTAIR acts a competing endogenous RNA to control the expression of notch3 via sponging miR-613 in pancreatic cancer. Oncotarget. 2017 May 16;8(20):32905-32917. eng. Epub 2017/04/19. doi:10.18632/oncotarget.16462.

52. Wong CH, Li CH, He Q, Chan SL, Tong JH, To KF, et al. Ectopic HOTTIP expression induces noncanonical transactivation pathways to promote growth and invasiveness in pancreatic ductal adenocarcinoma. Cancer letters. 2020 May 1;477:1-9. eng. Epub 2020/03/03. doi:10.1016/j.canlet.2020.02.038.

53. Yin F, Zhang Q, Dong Z, Hu J, Ma Z. LncRNA HOTTIP Participates in Cisplatin Resistance of Tumor Cells by Regulating miR-137 Expression in Pancreatic Cancer. OncoTargets and therapy. 2020;13:2689-2699. eng. Epub 2020/04/14. doi:10.2147/ott.S234924.

54. Takahashi K, Ota Y, Kogure T, Suzuki Y, Iwamoto H, Yamakita K, et al. Circulating extracellular vesicle-encapsulated HULC is a potential biomarker for human pancreatic cancer. Cancer science. 2020 Jan;111(1):98-111. eng. Epub 2019/11/13. doi:10.1111/cas.14232.

55. Pan HY, Mi YY, Xu K, Zhang Z, Wu H, Zhang W, et al. Association of C-reactive protein (CRP) rs1205 and rs2808630 variants and risk of cancer. Journal of cellular physiology. 2020 Nov;235(11):8571-8584. eng. Epub 2020/04/25. doi:10.1002/jcp.29701.

56. Xiong X, Shi Q, Yang X, Wang W, Tao J. LINC00052 functions as a tumor suppressor through negatively modulating miR-330-3p in pancreatic cancer. Journal of cellular physiology. 2019 Sep;234(9):15619-15626. eng. Epub 2019/02/04. doi:10.1002/jcp.28209.

57. Yuan ZJ, Yu C, Hu XF, He Y, Chen P, Ouyang SX. LINC00152 promotes pancreatic cancer cell proliferation, migration and invasion via targeting miR-150. Am J Transl Res. 2020;12(5):2241-2256. eng. Epub 2020/06/09.

58. Zhai S, Xu Z, Xie J, Zhang J, Wang X, Peng C, et al. Epigenetic silencing of LncRNA LINC00261 promotes c-myc-mediated aerobic glycolysis by regulating miR-222-3p/HIPK2/ERK axis and sequestering IGF2BP1. Oncogene. 2021 Jan;40(2):277-291. eng. Epub 2020/10/31. doi:10.1038/s41388-020-01525-3.

59. Wang X, Gao X, Tian J, Zhang R, Qiao Y, Hua X, et al. LINC00261 inhibits progression of pancreatic cancer by down-regulating miR-23a-3p. Archives of biochemistry and biophysics. 2020 Aug 15;689:108469. eng. Epub 2020/06/27. doi:10.1016/j.abb.2020.108469.

60. Zhang R, Hao S, Yang L, Xie J, Chen S, Gu G. LINC00339 promotes cell proliferation and metastasis in pancreatic cancer via miR-497-5p/IGF1R axis. Journal of BUON : official journal of the Balkan Union of Oncology. 2019 Mar-Apr;24(2):729-738. eng. Epub 2019/05/28.

61. Margue C, Philippidou D, Kozar I, Cesi G, Felten P, Kulms D, et al. Kinase inhibitor library screening identifies synergistic drug combinations effective in sensitive and resistant melanoma cells. Journal of experimental & clinical cancer research : CR. 2019 Feb 6;38(1):56. eng. Epub 2019/02/08. doi:10.1186/s13046-019-1038-x.

62. Wu J, Sun S, Liao W, Chen E, Wang X, Song Y, et al. LINC00460 promotes pancreatic cancer progression by sponging miR-491-5p. The journal of gene medicine. 2021 Jun;23(6):e3333. eng. Epub 2021/04/01. doi:10.1002/jgm.3333.

63. Cheng J, Lou Y, Jiang K. Downregulation of long non-coding RNA LINC00460 inhibits the proliferation, migration and invasion, and promotes apoptosis of pancreatic cancer cells via modulation of the miR-320b/ARF1 axis. Bioengineered. 2021 Dec;12(1):96-107. eng. Epub 2020/12/22. doi:10.1080/21655979.2020.1863035.

64. Zhou B, Guo W, Sun C, Zhang B, Zheng F. Linc00462 promotes pancreatic cancer invasiveness through the miR-665/TGFBR1-TGFBR2/SMAD2/3 pathway. Cell Death Dis. 2018 Jun 13;9(6):706. eng. Epub 2018/06/15. doi:10.1038/s41419-018-0724-5.

65. Zhou WY, Zhang MM, Liu C, Kang Y, Wang JO, Yang XH. Long noncoding RNA LINC00473 drives the progression of pancreatic cancer via upregulating programmed death-ligand 1 by sponging microRNA-195-5p. Journal of cellular physiology. 2019 Dec;234(12):23176-23189. eng. Epub 2019/06/18. doi:10.1002/jcp.28884.

66. Zhao X, Liu Y, Li Z, Zheng S, Wang Z, Li W, et al. Linc00511 acts as a competing endogenous RNA to regulate VEGFA expression through sponging hsa-miR-29b-3p in pancreatic ductal adenocarcinoma. J Cell Mol Med. 2018 Jan;22(1):655-667. eng. Epub 2017/10/07. doi:10.1111/jcmm.13351.

67. Han Q, Li J, Xiong J, Song Z. Long noncoding RNA LINC00514 accelerates pancreatic cancer progression by acting as a ceRNA of miR-28-5p to upregulate Rap1b expression. Journal of experimental & clinical cancer research : CR. 2020 Aug 8;39(1):151. eng. Epub 2020/08/11. doi:10.1186/s13046-020-01660-5.

68. Bi S, Wang Y, Feng H, Li Q. Long noncoding RNA LINC00657 enhances the malignancy of pancreatic ductal adenocarcinoma by acting as a competing endogenous RNA on microRNA-433 to increase PAK4 expression. Cell Cycle. 2020 Apr;19(7):801-816. eng. Epub 2020/03/03. doi:10.1080/15384101.2020.1731645.

69. Li T, Zhao H, Zhou H, Geng T. LncRNA LINC00857 strengthens the malignancy behaviors of pancreatic adenocarcinoma cells by serving as a competing endogenous RNA for miR-340-5p to upregulate TGFA expression. PloS one. 2021;16(3):e0247817. eng. Epub 2021/03/05. doi:10.1371/journal.pone.0247817.

70. Meng X, Deng Y, He S, Niu L, Zhu H. m(6)A-Mediated Upregulation of LINC00857 Promotes Pancreatic Cancer Tumorigenesis by Regulating the miR-150-5p/E2F3 Axis. Frontiers in oncology. 2021;11:629947. eng. Epub 2021/03/09. doi:10.3389/fonc.2021.629947.

71. Wang J, He Z, Xu J, Chen P, Jiang J. Long noncoding RNA LINC00941 promotes pancreatic cancer progression by competitively binding miR-335-5p to regulate ROCK1-mediated LIMK1/Cofilin-1 signaling. Cell Death Dis. 2021 Jan 4;12(1):36. eng. Epub 2021/01/09. doi:10.1038/s41419-020-03316-w.

72. Fang L, Wang SH, Cui YG, Huang L. LINC00941 promotes proliferation and metastasis of pancreatic adenocarcinoma by competitively binding miR-873-3p and thus upregulates ATXN2. European review for medical and pharmacological sciences. 2021 Feb;25(4):1861-1868. eng. Epub 2021/03/05. doi:10.26355/eurrev_202102_25081.

73. Chen S, Chen JZ, Zhang JQ, Chen HX, Qiu FN, Yan ML, et al. Silencing of long noncoding RNA LINC00958 prevents tumor initiation of pancreatic cancer by acting as a sponge of microRNA-330-5p to down-regulate PAX8. Cancer letters. 2019 Apr 1;446:49-61. eng. Epub 2019/01/15. doi:10.1016/j.canlet.2018.12.017.

74. Huang Y, Yan Q, Yu D, Sun X, Jiang S, Li W, et al. Long intergenic non-protein coding RNA 960 regulates cancer cell viability, migration and invasion through modulating miR-146a-5p/interleukin 1 receptor associated kinase 1 axis in pancreatic ductal adenocarcinoma. Bioengineered. 2021 Dec;12(1):369-381. eng. Epub 2021/01/01. doi:10.1080/21655979.2020.1868742.

75. Lei S, He Z, Chen T, Guo X, Zeng Z, Shen Y, et al. Long noncoding RNA 00976 promotes pancreatic cancer progression through OTUD7B by sponging miR-137 involving EGFR/MAPK pathway. Journal of experimental & clinical cancer research : CR. 2019 Nov 20;38(1):470. eng. Epub 2019/11/22. doi:10.1186/s13046-019-1388-4.

76. Zhu X, Niu X, Ge C. Inhibition of LINC00994 represses malignant behaviors of pancreatic cancer cells: interacting with miR-765-3p/RUNX2 axis. Cancer biology & therapy. 2019;20(6):799-811. eng. Epub 2019/02/12. doi:10.1080/15384047.2018.1564566.

77. Zhang L, Wang Y, Zhang L, You G, Li C, Meng B, et al. LINC01006 promotes cell proliferation and metastasis in pancreatic cancer via miR-2682-5p/HOXB8 axis. Cancer Cell Int. 2019;19:320. eng. Epub 2019/12/13. doi:10.1186/s12935-019-1036-2.

78. Pan S, Shen M, Zhou M, Shi X, He R, Yin T, et al. Long noncoding RNA LINC01111 suppresses pancreatic cancer aggressiveness by regulating DUSP1 expression via microRNA-3924. Cell Death Dis. 2019 Nov 25;10(12):883. eng. Epub 2019/11/27. doi:10.1038/s41419-019-2123-y.

79. Liu C, Wang JO, Zhou WY, Chang XY, Zhang MM, Zhang Y, et al. Long non-coding RNA LINC01207 silencing suppresses AGR2 expression to facilitate autophagy and apoptosis of pancreatic cancer cells by sponging miR-143-5p. Mol Cell Endocrinol. 2019 Aug 1;493:110424. eng. Epub 2019/04/17. doi:10.1016/j.mce.2019.04.004.

80. Cai M, Xu L, Shen L, Zhang J. [The expression of long non-coding RNA-LINC01410 in pancreatic cancer and its effect on proliferation and migration of pancreatic cancer cells]. Zhonghua yi xue za zhi. 2019 May 14;99(18):1406-1411. chi. Epub 2019/05/30. doi:10.3760/cma.j.issn.0376-2491.2019.18.010.

81. Zhai H, Zhang X, Sun X, Zhang D, Ma S. Long Non-coding RNA LINC01420 Contributes to Pancreatic Cancer Progression Through Targeting KRAS Proto-oncogene. Digestive diseases and sciences. 2020 Apr;65(4):1042-1052. eng. Epub 2019/09/29. doi:10.1007/s10620-019-05829-7.

82. Xu Z, Zhang D, Zhang Z, Luo W, Shi R, Yao J, et al. MicroRNA-505, Suppressed by Oncogenic Long Non-coding RNA LINC01448, Acts as a Novel Suppressor of Glycolysis and Tumor Progression Through Inhibiting HK2 Expression in Pancreatic Cancer. Front Cell Dev Biol. 2020;8:625056. eng. Epub 2021/02/02. doi:10.3389/fcell.2020.625056.

83. Lou C, Zhao J, Gu Y, Li Q, Tang S, Wu Y, et al. LINC01559 accelerates pancreatic cancer cell proliferation and migration through YAP-mediated pathway. Journal of cellular physiology. 2020 Apr;235(4):3928-3938. eng. Epub 2019/10/15. doi:10.1002/jcp.29288.

84. Chen X, Wang J, Xie F, Mou T, Zhong P, Hua H, et al. Long noncoding RNA LINC01559 promotes pancreatic cancer progression by acting as a competing endogenous RNA of miR-1343-3p to upregulate RAF1 expression. Aging. 2020 Jul 17;12(14):14452-14466. eng. Epub 2020/07/18. doi:10.18632/aging.103487.

85. Li K, Han H, Gu W, Cao C, Zheng P. Long non-coding RNA LINC01963 inhibits progression of pancreatic carcinoma by targeting miR-641/TMEFF2. Biomedicine & pharmacotherapy = Biomedecine & pharmacotherapie. 2020 Sep;129:110346. eng. Epub 2020/06/20. doi:10.1016/j.biopha.2020.110346.

86. Chen AY, Zhang K, Liu GQ. LncRNA LINP1 promotes malignant progression of pancreatic cancer by adsorbing microRNA-491-3p. European review for medical and pharmacological sciences. 2020 Sep;24(18):9315-9324. eng. Epub 2020/10/06. doi:10.26355/eurrev_202009_23013.

87. Gong Y, Dai HS, Shu JJ, Liu W, Bie P, Zhang LD. LNC00673 suppresses proliferation and metastasis of pancreatic cancer via target miR-504/ HNF1A. Journal of Cancer. 2020;11(4):940-948. eng. Epub 2020/01/18. doi:10.7150/jca.32855.

88. Liu Y, Guo C, Li F, Wu L. LncRNA LOXL1-AS1/miR-28-5p/SEMA7A axis facilitates pancreatic cancer progression. Cell biochemistry and function. 2020 Jan;38(1):58-65. eng. Epub 2019/11/17. doi:10.1002/cbf.3449.

89. Nai Y, Pan C, Hu X, Ma Y. LncRNA LUCAT1 contributes to cell proliferation and migration in human pancreatic ductal adenocarcinoma via sponging miR-539. Cancer medicine. 2020 Jan;9(2):757-767. eng. Epub 2019/12/04. doi:10.1002/cam4.2724.

90. Ye L, Feng W, Weng H, Yuan C, Liu J, Wang Z. MAFG-AS1 aggravates the progression of pancreatic cancer by sponging miR-3196 to boost NFIX. Cancer Cell Int. 2020 Dec 9;20(1):591. eng. Epub 2020/12/11. doi:10.1186/s12935-020-01669-y.

91. Zhuo M, Yuan C, Han T, Cui J, Jiao F, Wang L. A novel feedback loop between high MALAT-1 and low miR-200c-3p promotes cell migration and invasion in pancreatic ductal adenocarcinoma and is predictive of poor prognosis. BMC cancer. 2018 Oct 23;18(1):1032. eng. Epub 2018/10/26. doi:10.1186/s12885-018-4954-9.

92. Liu P, Yang H, Zhang J, Peng X, Lu Z, Tong W, et al. The lncRNA MALAT1 acts as a competing endogenous RNA to regulate KRAS expression by sponging miR-217 in pancreatic ductal adenocarcinoma. Scientific reports. 2017 Jul 12;7(1):5186. eng. Epub 2017/07/14. doi:10.1038/s41598-017-05274-4.

93. Zhang Y, Tang X, Shi M, Wen C, Shen B. MiR-216a decreases MALAT1 expression, induces G2/M arrest and apoptosis in pancreatic cancer cells. Biochem Biophys Res Commun. 2017 Feb 5;483(2):816-822. eng. Epub 2016/12/31. doi:10.1016/j.bbrc.2016.12.167.

94. Yang M, Sun S, Guo Y, Qin J, Liu G. Long non-coding RNA MCM3AP-AS1 promotes growth and migration through modulating FOXK1 by sponging miR-138-5p in pancreatic cancer. Molecular medicine (Cambridge, Mass). 2019 Dec 12;25(1):55. eng. Epub 2019/12/14. doi:10.1186/s10020-019-0121-2.

95. Han T, Zhuo M, Yuan C, Xiao X, Cui J, Qin G, et al. Coordinated silencing of the Sp1-mediated long noncoding RNA MEG3 by EZH2 and HDAC3 as a prognostic factor in pancreatic ductal adenocarcinoma. Cancer Biol Med. 2020 Nov 15;17(4):953-969. eng. Epub 2020/12/11. doi:10.20892/j.issn.2095-3941.2019.0427.

96. Li TF, Liu J, Fu SJ. The interaction of long non-coding RNA MIAT and miR-133 play a role in the proliferation and metastasis of pancreatic carcinoma. Biomedicine & pharmacotherapy = Biomedecine & pharmacotherapie. 2018 Aug;104:145-150. eng. Epub 2018/05/18. doi:10.1016/j.biopha.2018.05.043.

97. Qin Y, Liu X, Pan L, Zhou R, Zhang X. Long noncoding RNA MIR155HG facilitates pancreatic cancer progression through negative regulation of miR-802. J Cell Biochem. 2019 Oct;120(10):17926-17934. eng. Epub 2019/06/05. doi:10.1002/jcb.29060.

98. Yu T, Li G, Wang C, Gong G, Wang L, Li C, et al. MIR210HG regulates glycolysis, cell proliferation, and metastasis of pancreatic cancer cells through miR-125b-5p/HK2/PKM2 axis. RNA Biol. 2021 Jun 10:1-18. eng. Epub 2021/06/11. doi:10.1080/15476286.2021.1930755.

99. Yang H, Liu P, Zhang J, Peng X, Lu Z, Yu S, et al. Long noncoding RNA MIR31HG exhibits oncogenic property in pancreatic ductal adenocarcinoma and is negatively regulated by miR-193b. Oncogene. 2016 Jul 14;35(28):3647-57. eng. Epub 2015/11/10. doi:10.1038/onc.2015.430.

100. Sun Y, Wang P, Yang W, Shan Y, Zhang Q, Wu H. The role of lncRNA MSC-AS1/miR-29b-3p axis-mediated CDK14 modulation in pancreatic cancer proliferation and Gemcitabine-induced apoptosis. Cancer biology & therapy. 2019;20(6):729-739. eng. Epub 2019/03/28. doi:10.1080/15384047.2018.1529121.

101. Luo Z, Yi ZJ, Ou ZL, Han T, Wan T, Tang YC, et al. RELA/NEAT1/miR-302a-3p/RELA feedback loop modulates pancreatic ductal adenocarcinoma cell proliferation and migration. Journal of cellular physiology. 2019 Apr;234(4):3583-3597. eng. Epub 2018/10/27. doi:10.1002/jcp.27039.

102. Huang B, Liu C, Wu Q, Zhang J, Min Q, Sheng T, et al. Long non-coding RNA NEAT1 facilitates pancreatic cancer progression through negative modulation of miR-506-3p. Biochem Biophys Res Commun. 2017 Jan 22;482(4):828-834. eng. Epub 2016/11/27. doi:10.1016/j.bbrc.2016.11.120.

103. Li H, Wang X, Wen C, Huo Z, Wang W, Zhan Q, et al. Long noncoding RNA NORAD, a novel competing endogenous RNA, enhances the hypoxia-induced epithelial-mesenchymal transition to promote metastasis in pancreatic cancer. Molecular cancer. 2017 Nov 9;16(1):169. eng. Epub 2017/11/11. doi:10.1186/s12943-017-0738-0.

104. Li X, Deng SJ, Zhu S, Jin Y, Cui SP, Chen JY, et al. Hypoxia-induced lncRNA-NUTF2P3-001 contributes to tumorigenesis of pancreatic cancer by derepressing the miR-3923/KRAS pathway. Oncotarget. 2016 Feb 2;7(5):6000-14. eng. Epub 2016/01/13. doi:10.18632/oncotarget.6830.

105. Wu L, Liu Y, Guo C, Shao Y. LncRNA OIP5-AS1 promotes the malignancy of pancreatic ductal adenocarcinoma via regulating miR-429/FOXD1/ERK pathway. Cancer Cell Int. 2020;20:296. eng. Epub 2020/07/17. doi:10.1186/s12935-020-01366-w.

106. Li A, Feng L, Niu X, Zeng Q, Li B, You Z. Downregulation of OIP5-AS1 affects proNGF-induced pancreatic cancer metastasis by inhibiting p75NTR levels. Aging. 2021 Apr 3;13(7):10688-10702. eng. Epub 2021/04/07. doi:10.18632/aging.202847.

107. Meng X, Ma J, Wang B, Wu X, Liu Z. Long non-coding RNA OIP5-AS1 promotes pancreatic cancer cell growth through sponging miR-342-3p via AKT/ERK signaling pathway. Journal of physiology and biochemistry. 2020 May;76(2):301-315. eng. Epub 2020/03/12. doi:10.1007/s13105-020-00734-4.

108. Shi C, Zhang H, Wang M, Tian R, Li X, Feng Y, et al. OPA Interacting Protein 5 Antisense RNA 1 Expedites Cell Migration and Invasion Through FOXM1/ Wnt/β-Catenin Pathway in Pancreatic Cancer. Digestive diseases and sciences. 2021 Mar 29. eng. Epub 2021/03/31. doi:10.1007/s10620-021-06919-1.

109. Hu X, Zhang L, Tian J, Ma J. Long non-coding RNA PART1 predicts a poor prognosis and promotes the malignant progression of pancreatic cancer by sponging miR-122. World journal of surgical oncology. 2021 Apr 17;19(1):122. eng. Epub 2021/04/19. doi:10.1186/s12957-021-02232-3.

110. Wang W, Li X, Guan C, Hu Z, Zhao Y, Li W, et al. LncRNA PCAT6 promotes the proliferation, migration and invasion of pancreatic ductal adenocarcinoma via regulating miR-185-5p/CBX2 axis. Pathology, research and practice. 2020 Sep;216(9):153074. eng. Epub 2020/08/23. doi:10.1016/j.prp.2020.153074.

111. Zhang Y, Ma H, Chen C. Long non‑coding RNA PCED1B‑AS1 promotes pancreatic ductal adenocarcinoma progression by regulating the miR‑411‑3p/HIF‑1α axis. Oncology reports. 2021 Jul;46(1). eng. Epub 2021/05/27. doi:10.3892/or.2021.8085.

112. Zhang H, Zhu C, He Z, Chen S, Li L, Sun C. LncRNA PSMB8-AS1 contributes to pancreatic cancer progression via modulating miR-382-3p/STAT1/PD-L1 axis. Journal of experimental & clinical cancer research : CR. 2020 Sep 5;39(1):179. eng. Epub 2020/09/07. doi:10.1186/s13046-020-01687-8.

113. Liu W, Tang J, Zhang H, Kong F, Zhu H, Li P, et al. A novel lncRNA PTTG3P/miR-132/212-3p/FoxM1 feedback loop facilitates tumorigenesis and metastasis of pancreatic cancer. Cell death discovery. 2020 Nov 30;6(1):136. eng. Epub 2020/12/11. doi:10.1038/s41420-020-00360-5.

114. Zhou C, Yi C, Yi Y, Qin W, Yan Y, Dong X, et al. LncRNA PVT1 promotes gemcitabine resistance of pancreatic cancer via activating Wnt/β-catenin and autophagy pathway through modulating the miR-619-5p/Pygo2 and miR-619-5p/ATG14 axes. Molecular cancer. 2020 Jul 29;19(1):118. eng. Epub 2020/07/31. doi:10.1186/s12943-020-01237-y.

115. Huang F, Chen W, Peng J, Li Y, Zhuang Y, Zhu Z, et al. LncRNA PVT1 triggers Cyto-protective autophagy and promotes pancreatic ductal adenocarcinoma development via the miR-20a-5p/ULK1 Axis. Molecular cancer. 2018 Jul 12;17(1):98. eng. Epub 2018/07/14. doi:10.1186/s12943-018-0845-6.

116. Zhao L, Kong H, Sun H, Chen Z, Chen B, Zhou M. LncRNA-PVT1 promotes pancreatic cancer cells proliferation and migration through acting as a molecular sponge to regulate miR-448. Journal of cellular physiology. 2018 May;233(5):4044-4055. eng. Epub 2017/06/29. doi:10.1002/jcp.26072.

117. Sun J, Zhang P, Yin T, Zhang F, Wang W. Upregulation of LncRNA PVT1 Facilitates Pancreatic Ductal Adenocarcinoma Cell Progression and Glycolysis by Regulating MiR-519d-3p and HIF-1A. Journal of Cancer. 2020;11(9):2572-2579. eng. Epub 2020/03/24. doi:10.7150/jca.37959.

118. Yan J, Jia Y, Chen H, Chen W, Zhou X. Long non-coding RNA PXN-AS1 suppresses pancreatic cancer progression by acting as a competing endogenous RNA of miR-3064 to upregulate PIP4K2B expression. Journal of experimental & clinical cancer research : CR. 2019 Sep 5;38(1):390. eng. Epub 2019/09/07. doi:10.1186/s13046-019-1379-5.

119. Fu Z, Li G, Li Z, Wang Y, Zhao Y, Zheng S, et al. Endogenous miRNA Sponge LincRNA-ROR promotes proliferation, invasion and stem cell-like phenotype of pancreatic cancer cells. Cell death discovery. 2017;3:17004. eng. Epub 2017/06/06. doi:10.1038/cddiscovery.2017.4.

120. Li C, Zhao Z, Zhou Z, Liu R. Linc-ROR confers gemcitabine resistance to pancreatic cancer cells via inducing autophagy and modulating the miR-124/PTBP1/PKM2 axis. Cancer chemotherapy and pharmacology. 2016 Dec;78(6):1199-1207. eng. Epub 2016/10/28. doi:10.1007/s00280-016-3178-4.

121. Gao S, Wang P, Hua Y, Xi H, Meng Z, Liu T, et al. ROR functions as a ceRNA to regulate Nanog expression by sponging miR-145 and predicts poor prognosis in pancreatic cancer. Oncotarget. 2016 Jan 12;7(2):1608-18. eng. Epub 2015/12/05. doi:10.18632/oncotarget.6450.

122. Hua YQ, Zhu YD, Xie GQ, Zhang K, Sheng J, Zhu ZF, et al. Long non-coding SBF2-AS1 acting as a competing endogenous RNA to sponge microRNA-142-3p to participate in gemcitabine resistance in pancreatic cancer via upregulating TWF1. Aging. 2019 Nov 4;11(20):8860-8878. eng. Epub 2019/10/18. doi:10.18632/aging.102307.

123. Yin Z, Zhou Y, Ma T, Chen S, Shi N, Zou Y, et al. Down-regulated lncRNA SBF2-AS1 in M2 macrophage-derived exosomes elevates miR-122-5p to restrict XIAP, thereby limiting pancreatic cancer development. J Cell Mol Med. 2020 May;24(9):5028-5038. eng. Epub 2020/04/18. doi:10.1111/jcmm.15125.

124. Li D, Zhang X, Yang Y, Shen Y, Zhang Q, Liu H, et al. Long non-coding RNA SNHG1 promotes Cyclin D1-mediated proliferation in pancreatic cancer by acting as a ceRNA of miR-195. Int J Clin Exp Pathol. 2019;12(3):730-739. eng. Epub 2020/01/15.

125. Cao W, Zhou G. LncRNA SNHG12 contributes proliferation, invasion and epithelial-mesenchymal transition of pancreatic cancer cells by absorbing miRNA-320b. Bioscience reports. 2020 Jun 26;40(6). eng. Epub 2020/05/21. doi:10.1042/bsr20200805.

126. Deng PC, Chen WB, Cai HH, An Y, Wu XQ, Chen XM, et al. LncRNA SNHG14 potentiates pancreatic cancer progression via modulation of annexin A2 expression by acting as a competing endogenous RNA for miR-613. J Cell Mol Med. 2019 Nov;23(11):7222-7232. eng. Epub 2019/09/13. doi:10.1111/jcmm.14467.

127. Zhang X, Zhao P, Wang C, Xin B. SNHG14 enhances gemcitabine resistance by sponging miR-101 to stimulate cell autophagy in pancreatic cancer. Biochem Biophys Res Commun. 2019 Mar 19;510(4):508-514. eng. Epub 2019/02/10. doi:10.1016/j.bbrc.2019.01.109.

128. Liu S, Zhang W, Liu K, Liu Y. LncRNA SNHG16 promotes tumor growth of pancreatic cancer by targeting miR-218-5p. Biomedicine & pharmacotherapy = Biomedecine & pharmacotherapie. 2019 Jun;114:108862. eng. Epub 2019/04/14. doi:10.1016/j.biopha.2019.108862.

129. Xu H, Miao X, Li X, Chen H, Zhang B, Zhou W. LncRNA SNHG16 contributes to tumor progression via the miR-302b-3p/SLC2A4 axis in pancreatic adenocarcinoma. Cancer Cell Int. 2021 Jan 12;21(1):51. eng. Epub 2021/01/14. doi:10.1186/s12935-020-01715-9.

130. Yu Y, Dong JT, He B, Zou YF, Li XS, Xi CH, et al. LncRNA SNHG16 induces the SREBP2 to promote lipogenesis and enhance the progression of pancreatic cancer. Future oncology (London, England). 2019 Nov;15(33):3831-3844. eng. Epub 2019/10/31. doi:10.2217/fon-2019-0321.

131. Zhao L, Ye J, Lu Y, Sun C, Deng X. lncRNA SNHG17 promotes pancreatic carcinoma progression via cross-talking with miR-942. Am J Transl Res. 2021;13(3):1037-1050. eng. Epub 2021/04/13.

132. Zhang XX, Chen H, Li HY, Chen R, He L, Yang JL, et al. Long non-coding RNA small nucleolar RNA host gene 6 aggravates pancreatic cancer through upregulation of far upstream element binding protein 1 by sponging microRNA-26a-5p. Chinese medical journal. 2020 May 20;133(10):1211-1220. eng. Epub 2020/05/21. doi:10.1097/cm9.0000000000000758.

133. Cheng D, Fan J, Ma Y, Zhou Y, Qin K, Shi M, et al. LncRNA SNHG7 promotes pancreatic cancer proliferation through ID4 by sponging miR-342-3p. Cell & bioscience. 2019;9:28. eng. Epub 2019/04/06. doi:10.1186/s13578-019-0290-2.

134. Jian Y, Fan Q. Long non-coding RNA SNHG7 facilitates pancreatic cancer progression by regulating the miR-146b-5p/Robo1 axis. Experimental and therapeutic medicine. 2021 Apr;21(4):398. eng. Epub 2021/03/09. doi:10.3892/etm.2021.9829.

135. Li Z, Jiang P, Li J, Peng M, Zhao X, Zhang X, et al. Tumor-derived exosomal lnc-Sox2ot promotes EMT and stemness by acting as a ceRNA in pancreatic ductal adenocarcinoma. Oncogene. 2018 Jul;37(28):3822-3838. eng. Epub 2018/04/13. doi:10.1038/s41388-018-0237-9.

136. Li N, Yang G, Luo L, Ling L, Wang X, Shi L, et al. lncRNA THAP9-AS1 Promotes Pancreatic Ductal Adenocarcinoma Growth and Leads to a Poor Clinical Outcome via Sponging miR-484 and Interacting with YAP. Clinical cancer research : an official journal of the American Association for Cancer Research. 2020 Apr 1;26(7):1736-1748. eng. Epub 2019/12/14. doi:10.1158/1078-0432.Ccr-19-0674.

137. Xue F, Song X, Zhang S, Niu M, Cui Y, Wang Y, et al. Long non-coding RNA TMPO-AS1 serves as a tumor promoter in pancreatic carcinoma by regulating miR-383-5p/SOX11. Oncology letters. 2021 Apr;21(4):255. eng. Epub 2021/03/06. doi:10.3892/ol.2021.12517.

138. Zhang Y, Yang H, Du Y, Liu P, Zhang J, Li Y, et al. Long noncoding RNA TP53TG1 promotes pancreatic ductal adenocarcinoma development by acting as a molecular sponge of microRNA-96. Cancer science. 2019 Sep;110(9):2760-2772. eng. Epub 2019/07/22. doi:10.1111/cas.14136.

139. Wang B, Sun X, Huang KJ, Zhou LS, Qiu ZJ. Long non-coding RNA TP73-AS1 promotes pancreatic cancer growth and metastasis through miRNA-128-3p/GOLM1 axis. World J Gastroenterol. 2021 May 7;27(17):1993-2014. eng. Epub 2021/05/20. doi:10.3748/wjg.v27.i17.1993.

140. Miao H, Lu J, Guo Y, Qiu H, Zhang Y, Yao X, et al. LncRNA TP73-AS1 enhances the malignant properties of pancreatic ductal adenocarcinoma by increasing MMP14 expression through miRNA -200a sponging. J Cell Mol Med. 2021 Apr;25(7):3654-3664. eng. Epub 2021/03/09. doi:10.1111/jcmm.16425.

141. Cui XP, Wang CX, Wang ZY, Li J, Tan YW, Gu ST, et al. LncRNA TP73-AS1 sponges miR-141-3p to promote the migration and invasion of pancreatic cancer cells through the up-regulation of BDH2. Bioscience reports. 2019 Mar 29;39(3). eng. Epub 2019/01/16. doi:10.1042/bsr20181937.

142. Tasaki Y, Suzuki M, Katsushima K, Shinjo K, Iijima K, Murofushi Y, et al. Cancer-Specific Targeting of Taurine-Upregulated Gene 1 Enhances the Effects of Chemotherapy in Pancreatic Cancer. Cancer research. 2021 Apr 1;81(7):1654-1666. eng. Epub 2021/03/03. doi:10.1158/0008-5472.Can-20-3021.

143. Lu Y, Tang L, Zhang Z, Li S, Liang S, Ji L, et al. Long Noncoding RNA TUG1/miR-29c Axis Affects Cell Proliferation, Invasion, and Migration in Human Pancreatic Cancer. Disease markers. 2018;2018:6857042. eng. Epub 2019/01/01. doi:10.1155/2018/6857042.

144. Zhao L, Sun H, Kong H, Chen Z, Chen B, Zhou M. The Lncrna-TUG1/EZH2 Axis Promotes Pancreatic Cancer Cell Proliferation, Migration and EMT Phenotype Formation Through Sponging Mir-382. Cellular physiology and biochemistry : international journal of experimental cellular physiology, biochemistry, and pharmacology. 2017;42(6):2145-2158. eng. Epub 2017/08/17. doi:10.1159/000479990.

145. Yue L, Guo J. LncRNA TUSC7 suppresses pancreatic carcinoma progression by modulating miR-371a-5p expression. Journal of cellular physiology. 2019 Feb 4. eng. Epub 2019/02/05. doi:10.1002/jcp.28248.

146. Guo Z, Wang X, Yang Y, Chen W, Zhang K, Teng B, et al. Hypoxic Tumor-Derived Exosomal Long Noncoding RNA UCA1 Promotes Angiogenesis via miR-96-5p/AMOTL2 in Pancreatic Cancer. Molecular therapy Nucleic acids. 2020 Aug 25;22:179-195. eng. Epub 2020/09/18. doi:10.1016/j.omtn.2020.08.021.

147. Liu Y, Feng W, Gu S, Wang H, Zhang Y, Chen W, et al. The UCA1/KRAS axis promotes human pancreatic ductal adenocarcinoma stem cell properties and tumor growth. Am J Cancer Res. 2019;9(3):496-510. eng. Epub 2019/04/06.

148. Wu X, Li C, Mariyam Z, Jiang P, Zhou M, Zeb F, et al. Acrolein-induced atherogenesis by stimulation of hepatic flavin containing monooxygenase 3 and a protection from hydroxytyrosol. Journal of cellular physiology. 2018 Jan;234(1):475-485. eng. Epub 2018/06/29. doi:10.1002/jcp.26600.

149. Zhang X, Gao F, Zhou L, Wang H, Shi G, Tan X. UCA1 Regulates the Growth and Metastasis of Pancreatic Cancer by Sponging miR-135a. Oncol Res. 2017 Nov 2;25(9):1529-1541. eng. Epub 2017/03/21. doi:10.3727/096504017x14888987683152.

150. Zhou Y, Chen Y, Ding W, Hua Z, Wang L, Zhu Y, et al. LncRNA UCA1 impacts cell proliferation, invasion, and migration of pancreatic cancer through regulating miR-96/FOXO3. IUBMB Life. 2018 Apr;70(4):276-290. eng. Epub 2018/03/04. doi:10.1002/iub.1699.

151. Shen J, Hong L, Yu D, Cao T, Zhou Z, He S. LncRNA XIST promotes pancreatic cancer migration, invasion and EMT by sponging miR-429 to modulate ZEB1 expression. The international journal of biochemistry & cell biology. 2019 Aug;113:17-26. eng. Epub 2019/06/05. doi:10.1016/j.biocel.2019.05.021.

152. Wei W, Liu Y, Lu Y, Yang B, Tang L. LncRNA XIST Promotes Pancreatic Cancer Proliferation Through miR-133a/EGFR. J Cell Biochem. 2017 Oct;118(10):3349-3358. eng. Epub 2017/03/16. doi:10.1002/jcb.25988.

153. Sun Z, Zhang B, Cui T. Long non-coding RNA XIST exerts oncogenic functions in pancreatic cancer via miR-34a-5p. Oncology reports. 2018 Apr;39(4):1591-1600. eng. Epub 2018/02/03. doi:10.3892/or.2018.6245.

154. Zou L, Chen FR, Xia RP, Wang HW, Xie ZR, Xu Y, et al. Long noncoding RNA XIST regulates the EGF receptor to promote TGF-β1-induced epithelial-mesenchymal transition in pancreatic cancer. Biochem Cell Biol. 2020 Apr;98(2):267-276. eng. Epub 2019/04/24. doi:10.1139/bcb-2018-0274.

155. Sun J, Zhang Y. LncRNA XIST enhanced TGF-β2 expression by targeting miR-141-3p to promote pancreatic cancer cells invasion. Bioscience reports. 2019 Jul 31;39(7). eng. Epub 2019/06/20. doi:10.1042/bsr20190332.

156. Liang S, Gong X, Zhang G, Huang G, Lu Y, Li Y. The lncRNA XIST interacts with miR-140/miR-124/iASPP axis to promote pancreatic carcinoma growth. Oncotarget. 2017 Dec 26;8(69):113701-113718. eng. Epub 2018/01/27. doi:10.18632/oncotarget.22555.

157. Liu PJ, Pan YH, Wang DW, You D. Long non‑coding RNA XIST promotes cell proliferation of pancreatic cancer through miR‑137 and Notch1 pathway. European review for medical and pharmacological sciences. 2020 Dec;24(23):12161-12170. eng. Epub 2020/12/19. doi:10.26355/eurrev_202012_24005.

158. Wei G, Lu T, Shen J, Wang J. LncRNA ZEB1-AS1 promotes pancreatic cancer progression by regulating miR-505-3p/TRIB2 axis. Biochem Biophys Res Commun. 2020 Aug 6;528(4):644-649. eng. Epub 2020/06/10. doi:10.1016/j.bbrc.2020.05.105.

159. Gao H, Gong N, Ma Z, Miao X, Chen J, Cao Y, et al. LncRNA ZEB2-AS1 promotes pancreatic cancer cell growth and invasion through regulating the miR-204/HMGB1 axis. Int J Biol Macromol. 2018 Sep;116:545-551. eng. Epub 2018/05/13. doi:10.1016/j.ijbiomac.2018.05.044.

160. Liu J, Zhu Y, Ge C. LncRNA ZFAS1 promotes pancreatic adenocarcinoma metastasis via the RHOA/ROCK2 pathway by sponging miR-3924. Cancer Cell Int. 2020;20:249. eng. Epub 2020/06/20. doi:10.1186/s12935-020-01322-8.
